# Characterization of pressure-mediated vascular tone in resistance arteries from bile duct-ligated rats

**DOI:** 10.18632/oncotarget.15409

**Published:** 2017-02-16

**Authors:** Ravirajsinh N. Jadeja, Menaka C. Thounaojam, Sandeep Khurana

**Affiliations:** ^1^ Digestive Health Center, Medical College of Georgia, Augusta, GA, USA; ^2^ Department of Biochemistry and Cancer Biology, School of Medicine, Meharry Medical College, Nashville, TN, USA

**Keywords:** cirrhosis, portal hypertension, myogenic tone, vascular dysfunction, mesenteric arteries, Pathology Section

## Abstract

In cirrhosis, changes in pressure-mediated vascular tone, a key determinant of systemic vascular resistance (SVR), are unknown. To address this gap in knowledge, we assessed *ex vivo* dynamics of pressurized mesenteric resistance arteries (diameter ~ 260 μm) from bile duct-ligated (BDL) and sham-operated (SHAM) rats and determined the underlying mechanisms. At isobaric intraluminal pressure (70 mmHg) as well as with step-wise increase in pressure (10-110 mmHg), arteries from SHAM-rats constricted more than BDL-rats, and had reduced luminal area. In both groups, incubation with LNAME (a NOS inhibitor) had no effect on pressure-mediated tone, and expression of NOS isoforms were similar. TEA, which enhances Ca^2+^ influx, augmented arterial tone only in SHAM-rats, with minimal effect in those from BDL-rats that was associated with reduced expression of Ca^2+^ channel TRPC6. In permeabilized arteries, high-dose Ca^2+^ and γGTP enhanced the vascular tone, which remained lower in BDL-rats that was associated with reduced ROCK2 and pMLC expression. Further, compared to SHAM-rats, in BDL-rats, arteries had reduced collagen expression which was associated with increased expression and activity of MMP-9. BDL-rats also had increased plasma reactive oxygen species (ROS). In vascular smooth muscle cells *in vitro*, peroxynitrite enhanced MMP-9 activity and reduced ROCK2 expression. These data provide evidence that in cirrhosis, pressure-mediated tone is reduced in resistance arteries, and suggest that circulating ROS play a role in reducing Ca^2+^ sensitivity and enhancing elasticity to induce arterial adaptations. These findings provide insights into mechanisms underlying attenuated SVR in cirrhosis.

## INTRODUCTION

Cirrhosis is characterized by contrasting changes in intrahepatic and extra-hepatic vascular beds―intrahepatic vascular resistance increases while systemic vascular resistance (SVR) decreases [1, 2]. These hemodynamic changes lead to portal hypertension (PHT), and arterial hypotension, respectively. Increased intrahepatic vascular resistance contributes to development of complications such as gastro-esophageal varices and ascites, and at the same time, attenuated SVR not only reduces renal perfusion that promotes fluid retention, but also impedes our ability to treat those complications. For example, to reduce PHT and risk of variceal bleeding, β-blockers are used to impede the increase in portal blood inflow (β_2_-effect). However, due to the effect of β-blockers on systemic vascular beds, already reduced-SVR may decrease further. The resulting arterial under-filling further attenuates renal perfusion and enhances fluid retention [3]. Therefore, in patients with cirrhosis, arterial hypotension and reduced SVR are critical barriers to pharmacological approaches to reduce intrahepatic vascular resistance and treat PHT.

Hemodynamic changes in murine models of cirrhosis mimic those in humans. Previous studies indicated that in the arteries from cirrhotic animals, agonist-induced contractility is reduced [4–6]; the implicated mechanisms include increased vascular nitric oxide (NO) and carbon monoxide, reduced vascular smooth muscle (VSM) Ca^2+^ influx, and reduced expression of Ca^2+^ sensitization proteins [7, 8]. However, most previous assessments were made in conduit arteries (e.g. aorta or superior mesenteric arteries), which are not the primary determinants of SVR. These studies also relied on isometric techniques (wire-myography), results of which may differ substantially from those obtained by pressure-myography [9]. Therefore, despite these advances in our understanding of mechanisms underlying vascular changes in cirrhosis, little is known about changes in vascular dynamics of small resistance arteries.

Small resistance arteries (diameter < 300 μm) are major determinants of SVR. The resistance artery smooth muscle has an intrinsic ability to constrict in response to increase in intraluminal pressure [10]. This feature, which is independent of endothelium and neurohumoral input, is not only a key determinant of SVR but also of local blood flow and tissue perfusion. The mechanisms underlying pressure-mediated vascular tone involve intracellular Ca^2+^ influx via Ca^2+^ channels, and Ca^2+^ sensitization by Rho-associated protein kinase (ROCK) and protein kinase C (PKC) pathways [11]. We demonstrated previously that the small mesenteric arteries from rats, in isobaric conditions, develop stable vasoconstriction known as myogenic tone (MT), and with step-wise increase in intraluminal pressure, constrict, a phenomenon known as myogenic response (MR) [12, 13].

Both MT and MR are excellent surrogates for systemic vascular resistance (SVR). In cirrhosis, SVR and arterial blood pressures are reduced; however, changes in MT and MR are unknown. We *hypothesize* that in cirrhosis, MT and MR are attenuated. To address these gaps in knowledge, and test our hypothesis, we investigated *ex vivo* changes in pressure-mediated vascular tone in the 4^th^-order mesenteric resistance arteries from BDL-rats, a validated model of cirrhosis [8, 14–18], and investigated the underlying mechanisms.

## RESULTS

### In BDL-rats, myogenic tone and myogenic response are reduced

**Figure 1:**
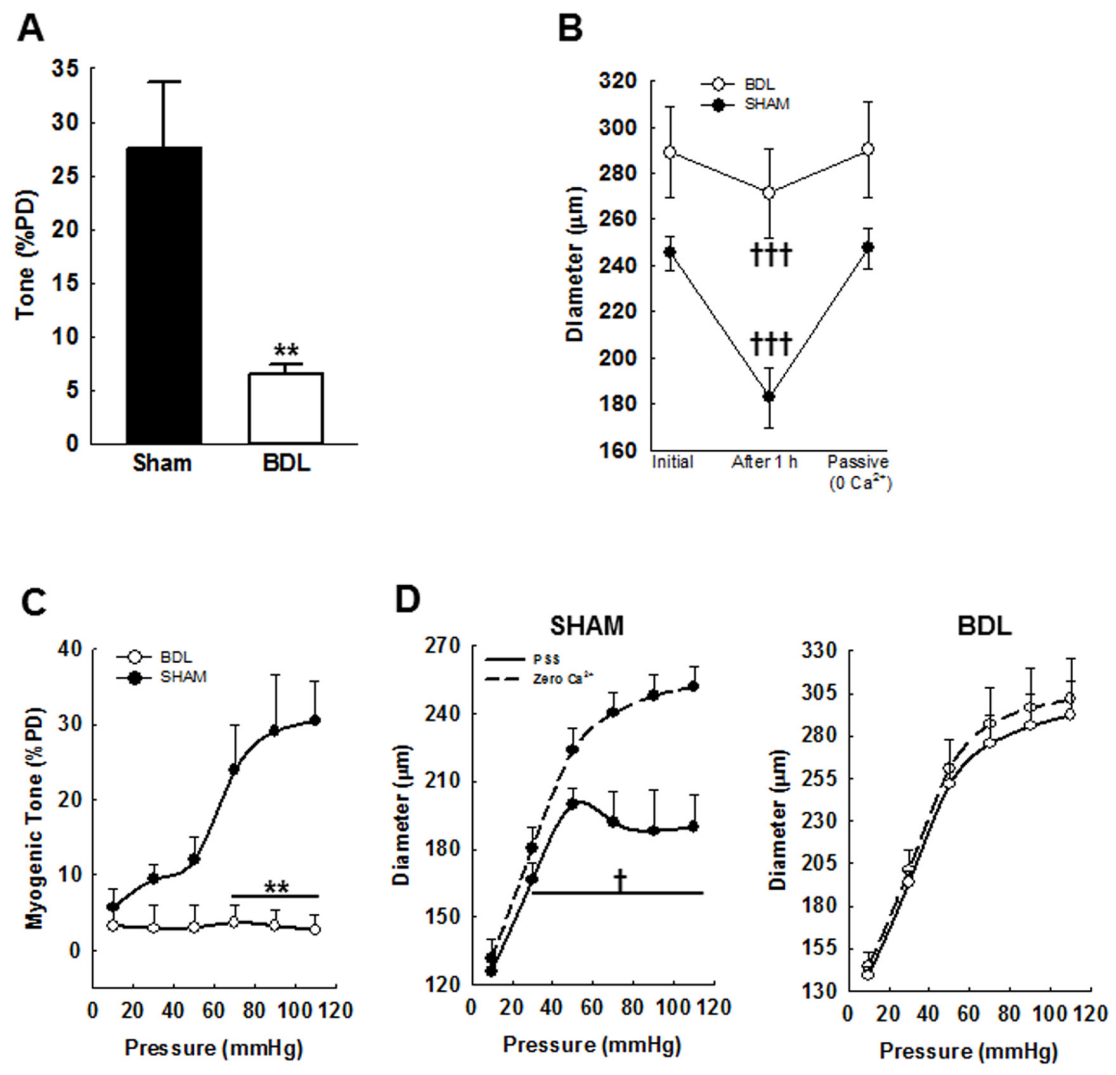
Effect of isobaric conditions and increasing intraluminal pressure on diameter of small mesenteric resistance arteries from SHAM- and BDL-rats **A**. Compared to SHAM-rats, fourth-order mesenteric arteries from BDL-rats pressurized at 70 mmHg and incubated with warm (37^ᵒ^C) PSS for 1 h developed reduced tone. **B**. Change in diameters of pressurized arterial segments are shown. Passive (maximal) diameter was determined by incubating the arteries in Ca^2+^-free PSS. **C**. To determine myogenic response, intraluminal pressure was increased in steps between 10-110 mmHg and spontaneous tone was allowed to develop until a stable diameter was achieved. The pressure-response was repeated in Ca^2+^-free PSS to determine the corresponding passive diameters. The myogenic tone is expressed as a percent of passive diameter (PD) and calculated as: (PD-achieved diameter)/PD×100. **D**. Arterial diameter at various pressure steps in PSS with and without Ca^2+^ are shown. **P < 0.01 when compared to SHAM-rats. † *P* < 0.05, ††† *P* < 0.001 when compared within treatment groups (*n* = 5-6/group).

Bile duct ligation in rats induces cirrhosis and development of ascites as outlined in supplementary material. The mesenteric arteries isolated from BDL- and SHAM-rats were pressurized at 70 mmHg and allowed to develop spontaneous constriction. As shown in Figure 1A, the arteries from the SHAM-rats constricted more than those from the BDL-rats (27 ± 6% *vs*. 6 ± 1%PD, respectively). Figure 1B shows changes in arterial diameter. To determine MR, the intraluminal pressure was increased in a step-wise manner (10-110 mmHg) and the arterial diameter recorded at each step. With increasing pressure, the arteries from the SHAM-rats constricted, however, those from the BDL-rats did not (Figures 1C & 1D). These data indicated that in the arteries from the BDL-rats, autoregulatory response was markedly attenuated.

### In BDL-rats, reduced myogenic response is independent of nitric oxide (NO)

**Figure 2:**
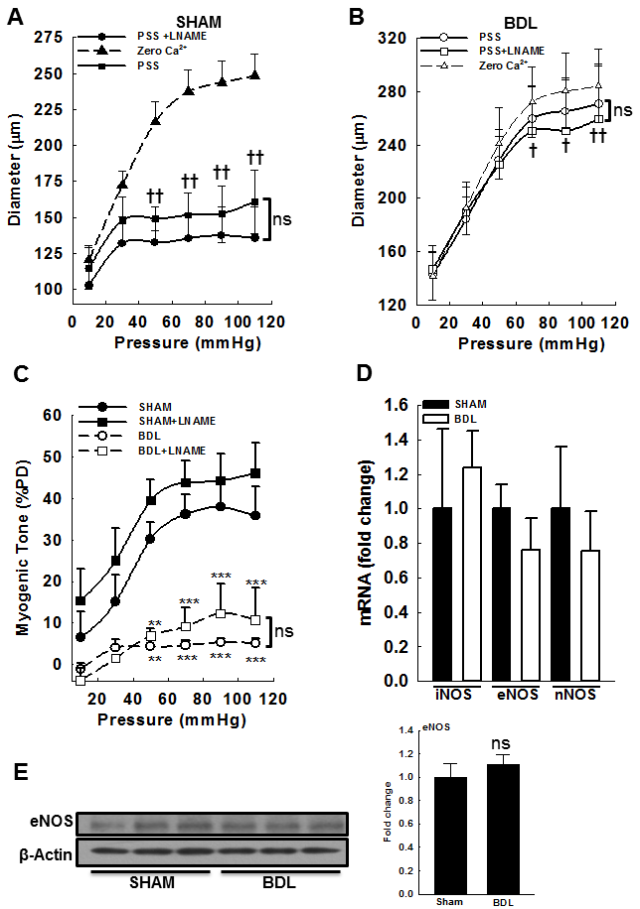
Effect of NOS inhibition on myogenic response in small mesenteric resistance arteries from SHAM- and BDL-rats Arteries were subjected to a series of intraluminal pressure steps between 10-110 mmHg before and after incubating with 300 μM LNAME (a NOS inhibitor for 60 min). The pressure-response was repeated in Ca^2+^-free PSS as described above. The arterial diameter at various pressure steps from **A**. SHAM-rats and **B**. BDL-rats are shown. **C**. Myogenic response was determined as described above. Incubation with LNAME improved MR minimally and non-significantly in both groups (*n* = 4-6/group). **D**. In mesenteric arteries from SHAM- and BDL-rats, the expression of mRNA for NOS isoforms was similar. **E**. The expression of NOS was also determined by immunoblotting; the immunoblots for eNOS and loading control are shown; The bar graph shows densitometry analysis. **P < 0.01, ***P < 0.001 when compared to SHAM-rats. † *P* < 0.05, †† *P* < 0.01 when compared within treatment groups; ns-not significant.

In cirrhosis, increased vascular NO is implicated in agonist-mediated hypocontractility as well increased acetylcholine-mediated relaxation [19]. Therefore, to assess the role of NO in pressure-mediated vascular tone, we assessed the role of NO in modulating MR, by incubating the arteries with 300 μM LNAME (a non-selective NOS inhibitor) for at least 1 h. Incubation of SHAM and BDL arteries with L-NAME led to no significant effect on the diameter when compared to non L-NAME treated groups respectively (Figures 2A & 2B). Further, pressure-induced myogenic tone in presence and absence of L-NAME remained similar in both groups (Figure 2C). In both groups, arterial expression of mRNA for NOS isoforms (iNOS, eNOS and nNOS) was similar (Figure 2D). The protein expression of eNOS was confirmed by immunoblotting (Figure 2E), however that of iNOS and nNOS was too small to quantify (data not shown). These results indicated that after BDL, there is no change in NOS expression in small mesenteric arteries. Further, in small mesenteric arteries, local NO has no role in modulating pressure-mediated vascular tone.

### In BDL-rats, agonist-mediated vasoconstriction is reduced

**Figure 3:**
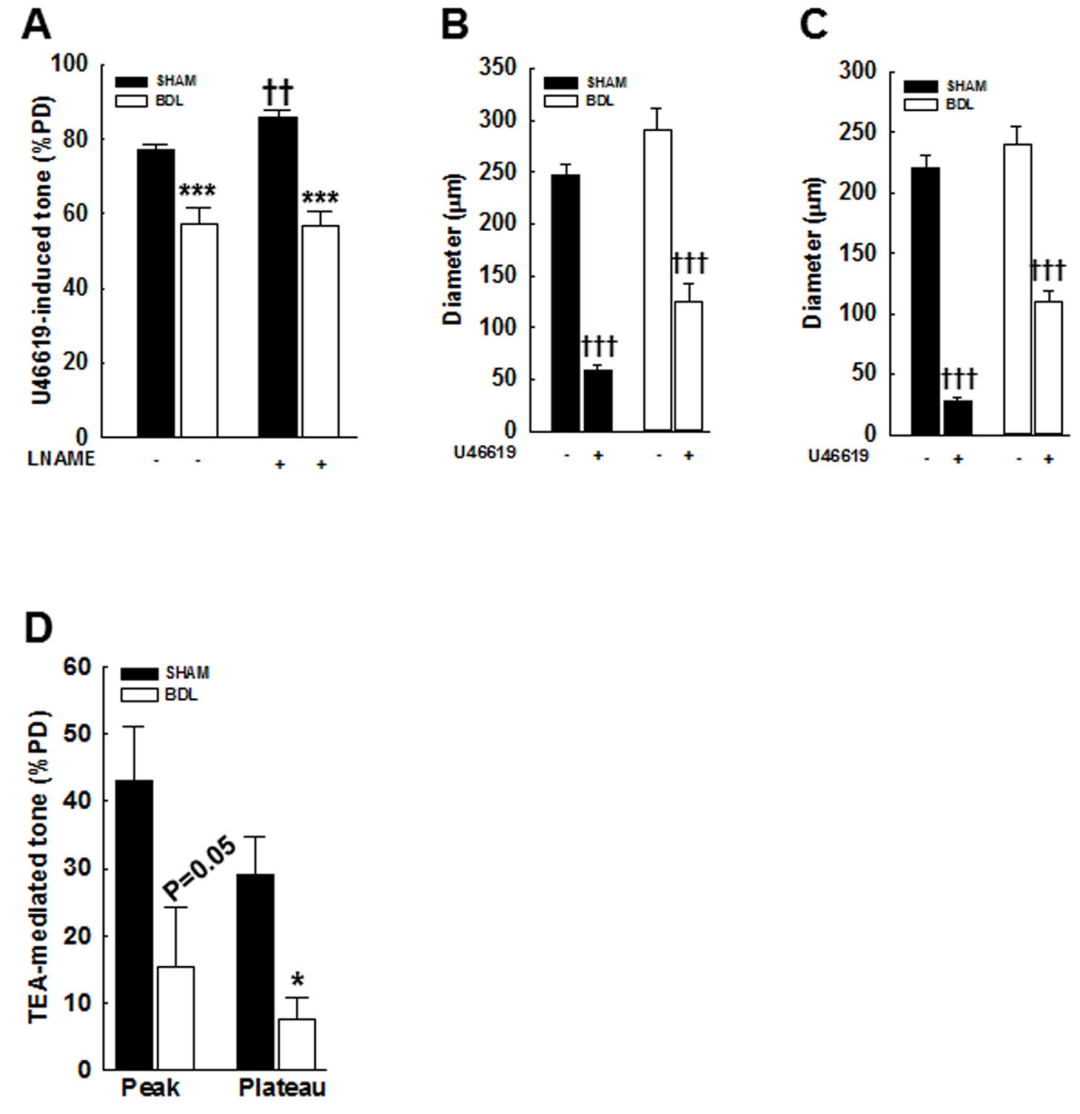
Effect of an agonist and TEA on vascular tone in pressurized mesenteric arteries from SHAM- and BDL-rats **A**. In the arteries from BDL-rats, U46619 (a thromboxane analogue and a ROCK activator)-mediated tone is reduced when compared to SHAM-rats. At 70 mmHg, arteries developed spontaneous vasoconstriction that was further augmented by 10 μM U46619. Incubation with 300 μM LNAME for 60 min (a NOS inhibitor) enhanced U46619-mediated vascular tone in the SHAM-rats only and had no effect in those from the BDL-rats. Arterial diameters in the **B**. absence and **C**. presence of LNAME are shown (*n* = 5-6/group). **D**. TEA (5 mM, a Ca^2+^-activated K^+^ channel blocker) enhanced vascular tone in pressurized arteries from the SHAM-rats and had minimal effect on those from the BDL-rats; peak and plateau responses are shown (*n* = 4-6/group). Effect of TEA was determined in arteries incubated with LNAME. *P < 0.05, ***P < 0.001 when compared to SHAM-rats. †† *P* < 0.01, ††† *P* < 0.001 when compared within treatment groups.

In the pressurized arteries from BDL-rats, 10 μM U46619 (a synthetic thromboxane A2 receptor agonist [20, 21])-mediated constriction was reduced compared to SHAM-rats (57.4±4.4 *vs*. 77±1.4%PD, Figure 3A). Incubation with 300 μM L-NAME for 60 min enhanced U46619-mediated constriction only in the SHAM-rats and had no effect in the BDL-rats (Figure 3A). Changes in arterial diameter are shown in Figures 3B & 3C. These results a) validated that in the BDL-model of cirrhosis, agonist-induced constriction is reduced and b) indicated that in normal physiological state, there is a possible interactions between NO and agonist-induced signaling, which is lost in cirrhosis [22].

Collectively, the experiments shown in Figures 2 and 3 indicate that in small mesenteric resistance arteries, NO reduces agonist (U46619)-mediated vascular tone, but has no role in pressure-mediated vascular tone. However, in cirrhosis, the effect of NO on agonist (U46619)-mediated vascular tone is lost, and the effect on pressure-mediated vascular tone still remains insignificant. Previous studies used agonists (vasoconstrictors) to demonstrate changes in vascular tone in cirrhosis. Our data demonstrate that NO has limited role in pressure-mediated vascular tone, and there is a disparity in how NO effects agonist- *vs*. pressure-mediated vasoconstriction.

### TEA has minimal effect on vascular tone in BDL-rats

**Figure 4:**
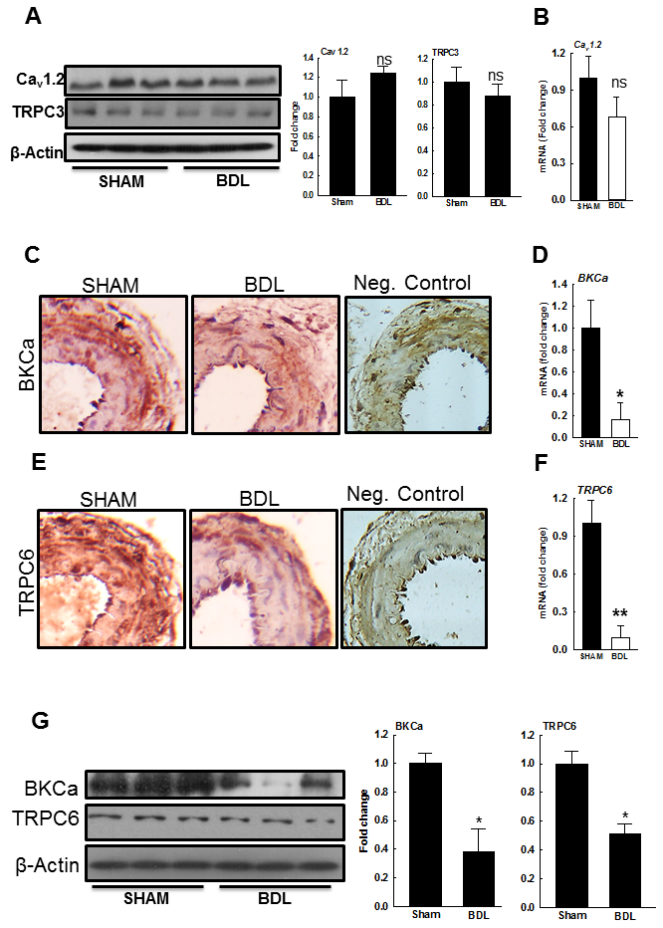
Expression of ion channels in small mesenteric resistance arteries from SHAM- and BDL-rats **A**. Immunoblots for Ca_v_1.2, TRPC3 and β-actin (loading control) from small mesenteric artery homogenates indicate no differences among both groups. The bar graphs show densitometry analyses for Ca_v_1.2 and TRPC3. **B**. qPCR for Ca_v_1.2. Representative IHC photographs and qPCR indicate that in the arteries from the BDL-rats, expressions of **C**., **D**. BK_Ca_ and **E**., **F**. TRPC6 are reduced when compared to SHAM-rats (*n* = 3-5/group). **G**. The immunoblots for both BK_Ca_ and TRPC6 and their loading controls are shown. The bar graphs show densitometry analyses for BK_Ca_ and TRPC6 (*n* = 3/group). *P < 0.05, **P < 0.01 when compared to SHAM-rats.

Tetraethyl ammonium (TEA, a Ca^2+^-activated K^+^ channel blocker) depolarizes vascular smooth muscle cells (VSMCs) and enhances Ca^2+^ influx [23]. TEA (5 mM) increased vascular tone only in pressurized arteries from the SHAM-rats and had minimal effect in those from the BDL-rats (Figure 3D). Since, the large-conductance Ca^2+^-activated K^+^ (BK_Ca_) channels inhibit the L-type voltage-dependent Ca^2+^ channels (VDCC), key channels that transports Ca^2+^ into VSMCs [24, 25], we assessed arterial expression of BK_Ca_ and Ca_v_1.2―the key subunit of VDCC. We observed no significant change in Ca_v_1.2 expression however; BK_Ca_ expression was reduced in arteries from the BDL-rats when compared to the SHAM-rats (Figures 4A-4D, 4G). These results suggested that the reduced BK_Ca_ expression, and possibly function, are the consequences of reduced vascular tone and not the cause for it. Therefore, we assessed the expression of other key channels that mediate Ca^2+^ influx.

### In BDL-rats, arterial expression of TRPC6 is reduced

Various ion channels alter [Ca^2+^] in VSMCs to modulate pressure-mediated tone. The role of such channels is being actively investigated, and their list continues to grow. Here we assessed the expression of transient receptor potential cation channels subtype 3 and 6 (TRPC3 and TRPC6), the key channels that regulate pressure-induced vasoconstriction in small resistance arteries [26, 27]. There was no significant change in TRPC3 expression (Figure 4A) however, TRPC6 expression was reduced in the arteries from BDL-rats when compared to SHAM-rats (Figures 4E-4G).

### In BDL-rats, mesenteric arteries have reduced sensitivity to Ca^2+^

**Figure 5:**
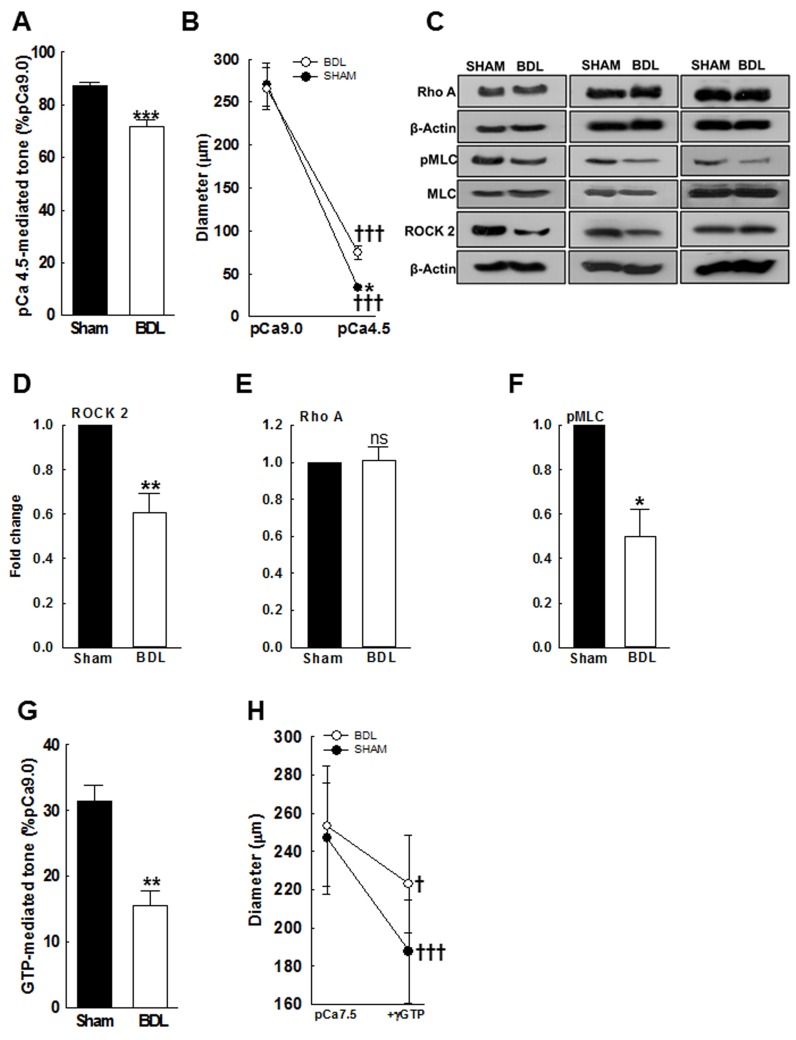
Ca^2+^ sensitivity of small mesenteric arteries from SHAM- and BDL-rats In β-escin-permeabilized arteries pressurized at 70 mmHg, the effect of high-dose Ca^2+^ was assessed. **A**. In response to pCa4.5, vascular tone increased in all rats but remained lower in the BDL-rats compared to SHAM-rats. **B**. Arterial diameters at pCa9.0 and pCa4.5 are shown (*n* = 4-5/group). **C**. Immunoblots indicate reduced arterial ROCK2 and pMLC expression in the BDL-rats (*n* = 3/group). **D**.-**F**. Summary data for ROCK2, RhoA and pMLC. **G**. Compared to SHAM-rats, permeabilized arteries from the BDL-rats developed reduced tone when stimulated with 100 μM γGTP (in pCa7.5). **H**. Arterial diameters before and after incubation with γGTP are shown (*n* = 4-5/group). *P < 0.05, **P < 0.01, ***P < 0.001 when compared to SHAM-rats. † *P* < 0.05, ††† *P* < 0.001 when compared within treatment groups.

To determine if bypassing the ion channels that regulate Ca^2+^ influx would restore vascular tone, we used permeabilized arterial preparations. As shown in Figure 5A, in the permeabilized arteries pressurized at 70 mmHg, high-dose Ca^2+^ (pCa4.5) increased vascular tone, which remained lower in the BDL-rats when compared to the SHAM-rats; respective changes in arterial diameter are shown in Figure 5B.

### In mesenteric arteries from BDL-rats, RhoA kinase pathway is inhibited

ROCK signaling is important in regulating VSM contractility and pressure-mediated tone [21]. The RhoA-mediated activation of ROCK2 regulates VSM contraction by modulating myosin light chain (MLC) phosphorylation. And, VSM contractility can be sustained or enhanced at fixed Ca^2+^ concentrations by increasing Ca^2+^ sensitivity via activation of ROCK. Therefore, in the mesenteric arteries from both groups, we evaluated the expression of RhoA, ROCK2 and phosphorylation of MLC subunit-20. Figures 5C-5F indicate that arterial expressions of ROCK2 and pMLC were decreased in the BDL-rats when compared to the SHAM-rats; The RhoA expression was similar. Based on these observations, we hypothesized that impaired Ca^2+^ sensitivity plays a significant role in reduced pressure-induced vascular tone in cirrhosis. Therefore, to assess the functional impact of low ROCK2 expression, in pressurized permeabilized arteries we assessed the effect of GTP, which activates ROCK. γGTP (100 μM)-mediated vascular tone was attenuated in the BDL-rats when compared to the SHAM-rats (Figures 5G & 5H).

### In BDL-rats, arterial intraluminal cross-section area and elasticity are increased

**Figure 6:**
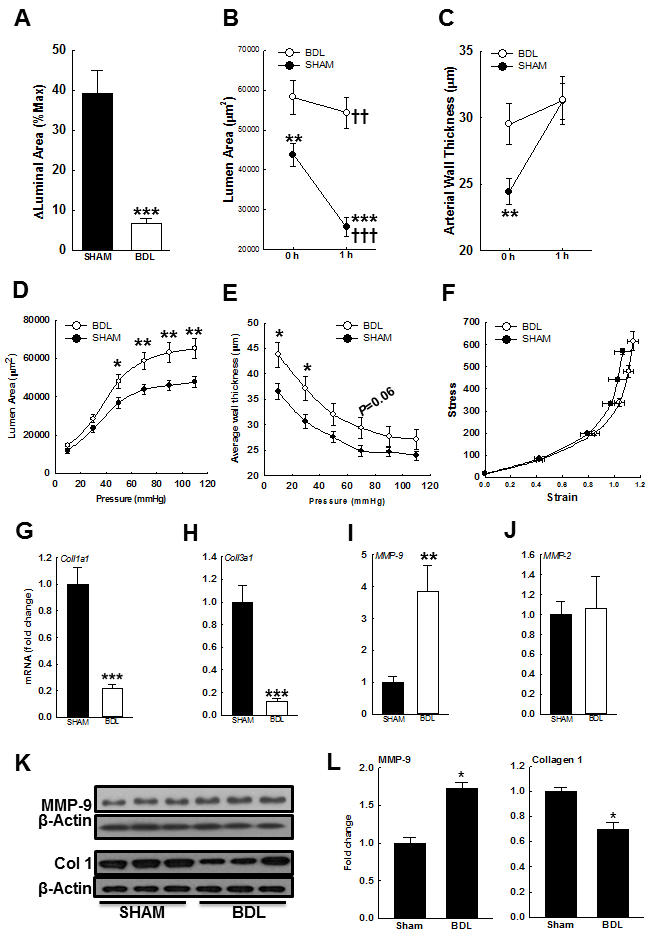
Effect of BDL on arterial elasticity **A**. After development of MT in isobaric conditions (within 1 h), the luminal area decreased in both but significantly more in the arteries from SHAM-rats than BDL-rats; **B**. Respective changes in luminal area. C. Arterial wall thickness was more in the BDL-rats, but after development of MT, was equal to those in the SHAM-rats (*n* = 16-17/group). To determine the effect of pressure on passive distension, arteries were incubated in Ca^2+^-free PSS. **D**. Increasing intraluminal pressure led to enhanced arterial distension in the BDL-rats. **E**. Arterial wall thickness in Ca^2+^-free PSS across intraluminal pressure range is shown. **F**. In the arteries from BDL-rats, stress-strain relation curve shifted right- and up-ward (*n* = 16/group). Arterial expression of mRNA for **G**. collagen 1a1, **H**. collagen 3a1, **I**. MMP-9 and **J**. MMP-2 are shown (*n* = 5-6/group). **K**. The immunoblots for both MMP-9, collagen 1 and their loading controls are shown. **L**. The bar graphs show densitometry analyses for MMP-9 and collagen 1 (*n* = 3/group). *P < 0.05, **P < 0.01, ***P < 0.001 when compared to SHAM-rats. †† *P* < 0.01, ††† *P* < 0.001 when compared within treatment groups.

**Figure 7:**
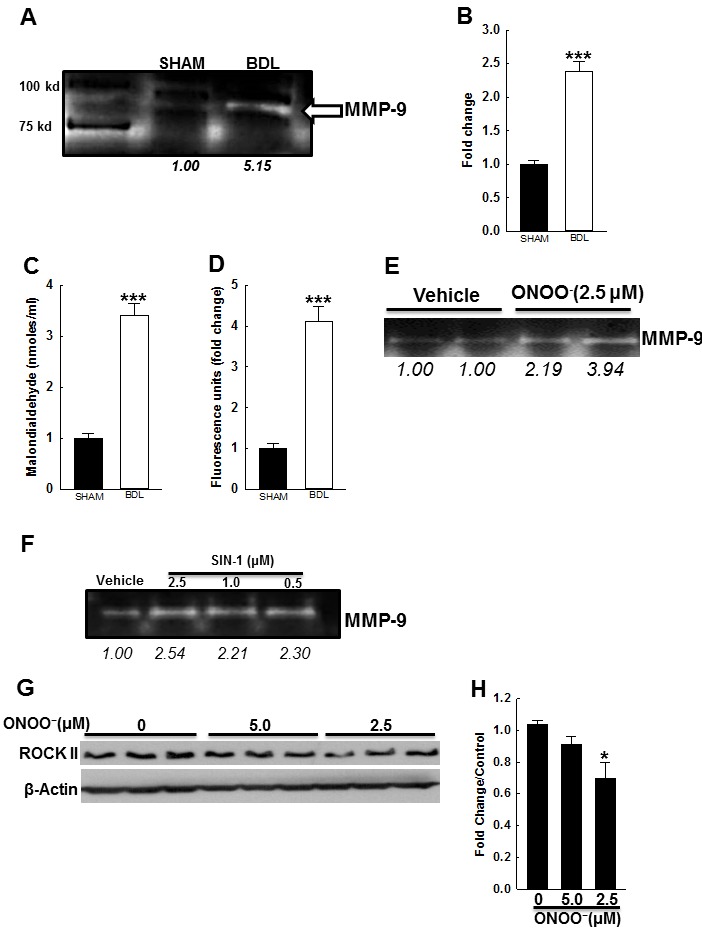
Effect of BDL and ROS on vascular MMP-9 activity **A**. Representative gelatin zymography indicates that in arteries from BDL-rats, MMP-9 activity is increased. **B**. Summary data (*n* = 3/group). Circulating ROS levels were measured in the plasma from BDL- and SHAM-rats. In BDL-rats, the plasma **C**. MDA levels and **D**. DCDF fluorescence were markedly increased when compared to SHAM-rats (*n* = 4/group). These data indicated that in cirrhosis, systemic circulation is an ample source of oxidative stress. Representative zymography indicates that incubation of rat VSMCs with both **E**. peroxynitrite and **F**. SIN-1 induces MMP-9 activity. **G**. Immunoblots and their densitometry data (I) indicate that in rat VSMCs, peroxynitrite reduces ROCK2 expression (*n* = 3/group). *Arabic numerals* indicate densitometric values. *P < 0.05 when compared to vehicle-treated cells. ***P < 0.001 when compared to SHAM-rats.

As shown in Figures 6A & 6B, the luminal area of arteries from the BDL-rats was more than those from the SHAM-rats. After development of MT, arterial luminal area reduced more in the SHAM-rats than in the BDL-rats. However, after development of MT, there was no difference in the arterial wall thickness among both groups (Figure 6C). Collectively these data suggested that factors other than Ca^2+^ influx and sensitization may be at play. Therefore, we assessed for changes in arterial distension in Ca^2+^-free PSS. At lower intraluminal pressure (10-30 mmHg), the luminal area was similar in both groups but at higher pressure (50-110 mmHg), was significantly higher in the BDL-rats (Figure 6D). Across the entire intraluminal pressure range, the BDL-rats had increased arterial wall thickness when compared to the SHAM-rats (Figure 6E). Additionally, in the BDL-rats, stress-strain curve shifted rightward (Figure 6F). These data indicated that the arteries from BDL-rats are more elastic that those from SHAM-rats. Therefore, to investigate the causes for increased arterial elasticity in the BDL-rats, we assessed for changes in modulators of extracellular matrix (ECM). In the BDL-rats, arterial expressions of collagen 1a1 and 3a1 were reduced and expression of MMP-9 increased (Figures 6G-6L). There was no change in MMP-2 expression (Figure 6J). Gelatin zymography demonstrated that in the arteries from BDL-rats, MMP-9 activity was increased when compared to SHAM-rats (Figures 7A&7B).

### In BDL-rats, plasma ROS levels are increased

In cirrhosis, numerous factors, i.e., inflammatory cytokines, shear stress and ROS, are thought to mediate vascular changes. For example, in conduit arteries of cirrhotic animals, peroxynitrite adducts are increased [14, 28]. However, it's not clear if peroxynitrite generation in small resistance arteries plays a role. We assessed for peroxynitrite adducts in the arteries from both groups (by IHC and immunoblotting) however, did not observe any substantive differences (data not shown). Since exogenous peroxynitrite has been shown to reduce vascular tone in cerebral arteries [29], we hypothesized that the vascular changes observed in the BDL-rats could be due to increased circulating ROS levels. We assessed in the rat plasma, ROS levels by measuring MDA levels, and oxidation of DCFDA to DCF. Figures 7C & 7D indicate that the BDL-rats had markedly increased circulating ROS levels compared to the SHAM-rats.

### In VSMCs, oxidative stress enhances MMP-9 activity and reduces ROCK2 expression

Since the circulating ROS levels were increased in the BDL-rats, we hypothesized that ROS induce molecular changes observed in resistance mesenteric arteries. To test this hypothesis we incubated the rat VSMCs with peroxynitrite (or SIN-1) and assessed MMP-9 activity in their supernatant. Figures 7E & 7F show that peroxynitrite (and SIN-1) enhanced MMP-9 activity in VSMC media. Finally, we assessed the effect of peroxynitrite on ROCK2 expression. We observed that rat VSMCs incubated with peroxynitrite for 24 h, had reduced ROCK2 expression (Figures 7G & 7H). These data indicated that in cirrhosis, increased circulating ROS potentially modulate arterial ECM, Ca^2+^ sensitivity, and thus elasticity.

## DISCUSSION

In early cirrhosis, increased cardiac output maintains normal blood pressure. As the cirrhosis progresses, systemic arterial vasodilation increases and SVR decreases, and increased cardiac output cannot compensate to maintain the “normal” blood pressure [30, 31]. The progressively decreasing SVR worsens renal under-perfusion and promotes fluid retention [3]. These hemodynamic changes trigger activation of renin-angiotensin system and sympathetic activity. However, despite elevated catecholamine and angiotensin levels, the arterial contractility remains attenuated [14, 32, 33]. Previous investigations identified various mechanisms likely responsible for hypocontractility of agonist-stimulated conduit arteries however, changes in pressure-mediated vascular tone have not been investigated.

MT and MR—the phenomena observed only in small resistance arteries and not in conduit arteries [25]—are independent of neurohumoral and endothelial contribution [11, 34]. The changes in the sensitivity of conduit arteries to vasoconstrictors and vasodilators, are not sufficient to explain the hemodynamic changes in cirrhosis. Therefore, in this study, we characterized the changes in pressure-mediated vascular tone of small resistance arteries, the key site for SVR.

We observed that in small resistance arteries from BDL-rats: 1) the pressure-mediated vascular tone is markedly attenuated that is independent of NO; 2) expression of TRPC6 is reduced; 3) expression of ROCK2 is reduced which is associated with reduced MLC phosphorylation, and responsible for attenuated GTP-mediated tone; 4) intraluminal cross-sectional area is increased that is associated with reduced expression of collagen and increased expression and activity of MMP-9; 5) circulating levels of ROS are augmented; and finally, in VSMCs, oxidative stress induces MMP-9 activity and reduces ROCK2 expression.

Endothelium-derived NO plays an important role in vascular reactivity by diffusing into VSMCs and reducing Ca^2+^ influx, thereby reducing vascular tone [35, 36]. Some studies showed that in conduit arteries from cirrhotic animals, acetylcholine-mediated vasodilation was augmented due to increase NO release; However, others suggested that NO's contribution was limited [14, 28]. We evaluated the role of NO by assessing for pressure-mediated vasoconstriction in the presence of a NOS inhibitor (LNAME). LNAME failed to correct MR in arteries from BDL-rats, or enhance MR in the arteries from SHAM-rats. Lack of change in expression of NOS isoforms corroborated our functional studies. Since the circulatory NO levels are elevated in cirrhosis [37], it is possible that increased NO produced in the conduit arteries may still circulate downstream to modulate vascular tone in the small resistance arteries.

The ion channels in VSMCs play major role in modulating pressure-mediated vascular tone [38].We assessed the expression of BK_Ca_, Ca_v_1.2, and TRPC3&6 in the arteries from BDL- and SHAM-rats. The arterial expression of TPRC3 and Ca_v_ 1.2 were not altered, while TRPC6 expression was significantly reduced in the BDL-rats. Since TRPC6 plays an important role in regulating pressure-mediated tone in resistant arteries [39], the observed reduction in vascular tone BDL-rats can be partially due to reduced expression of TRPC6. Concomitantly, BK_Ca_ expression was reduced in the arteries from BDL-rats. Since, BK_Ca_ inhibits VDCC, reduced expression of BK_Ca_ is expected to enhance pressure-mediated vascular tone. Interestingly, others also reported reduced expression of Ca^2+^-activated K^+^ channels in conduit arteries from cirrhotic rats [28]. Therefore, the reduced TEA response in the BDL-rats (Figure 4D) suggests that the reduced BK_Ca_ expression is an adaptive consequence to reduced MT and MR. TEA is a non-selective K_Ca_ channel blocker. In addition to BK_Ca_, it can also block other K_Ca_ channels. Moreover, various other K channels such as K_IR_, K_ATP_, and K_V_ which are also expressed in vasculature, can modulate vascular tone. In fact, there has been a suggestion that increased K_ATP_ gating may play role in reducing tone [40]. However, this was reported in VSMC from superior mesenteric arteries and not the resistance arteries. Therefore, in cirrhosis, further investigation is needed to assess the role of various K channels in regulation of vascular tone in resistance arteries.

Based on varied expression of ion channels, we hypothesized that bypassing them would restore pressure-mediated vascular tone in BDL-rats. To test this hypothesis, we used permeabilized arteries to achieve desired intracellular Ca^2+^ levels [41, 42]. High-dose Ca^2+^ enhanced vascular tone, which remained lower in BDL-rats. In conduit arteries from cirrhotic rats, expression of Ca^2+^ sensitization proteins is reduced which may be responsible for reduced agonist-induced constriction [8]. We observed that in arteries from BDL-rats, expression of ROCK2 was reduced. Reduced vasoconstriction in BDL-rats to stimulation by U46619, which activates ROCK pathway in VSMC [21, 43], also suggested reduced ROCK signaling [22, 44]. Importantly, attenuated vascular tone in response to exogenous GTP indicated that reduced expression of ROCK2 is functionally responsible for reduced pressure-mediated vascular tone in BDL-rats.

Resch and Wiest et al., suggested that the small mesenteric resistance arteries undergo remodeling that results in increased elasticity [45]. They observed in cirrhotic rats, an increase in luminal cross-sectional area and structural changes in elastic lamina but no change in ECM or vascular smooth muscle. We observed that in Ca^2+^-free PSS, the arteries from BDL-rats had increased wall thickness (Figure 7E), though in PSS with Ca^2+^ at 70 mmHg, they achieved similar thickness as SHAM-rats (Figure 7C). However, in both PSS with or without Ca^2+^, the cross-sectional area of arteries from BDL-rats remained higher. These data suggested that in BDL-rats, additional non-VSM factors are responsible for reduced vascular tone. Collagen is a known substrate of MMP-9 [46]. Reduced collagen expression, and increased MMP-9 expression and activity explain increased arterial elasticity in the BDL-rats as in Ca^2+^-free PSS, arteries from BDL-rats compared to those from SHAM-rats had increased distension (passive diameter) across all intraluminal pressures. Our findings match those of Resch and Wiest et al., but they are in contrast to those by Fernández-Varo et al [47], who reported a decrease in arterial wall thickness, increased collagen expression and reduced MMP-9 expression and activity in cirrhotic rats. These differences are potentially due to dissimilarities in mode of induction of cirrhosis (BDL *vs*. CCL_4_ + phenobarbital), arterial beds studied (resistance *vs*. conduit), and techniques used to measure arterial wall thickness (pressurized *ex vivo* *vs*. fixed).

Others have shown increased peroxynitrite staining in conduit arteries from cirrhotic rats [14, 28], however, in small resistance arteries we could not replicate these results (not shown). We measured ROS levels in systemic circulation from BDL- and SHAM-rats, and their role in MMP-9 activity. Our findings indicate that circulatory ROS levels are markedly increased in BDL-rats, and in VSMCs *in vitro*, ROS increased MMP-9 activity and reduced ROCK2 expression (Figure 7). These data indicated that systemic circulation is a source of stimuli that reduces arterial tone by possibly ECM remodeling and reducing Ca^2+^ sensitivity.

Our study has a few limitations. Despite decrease in TRPC6 expression, we did not measure associated decrease in Ca^2+^ influx. The techniques and instruments required to simultaneously measure changes in MT and Ca^2+^ influx in small arteries are highly challenging and not commonly available. Additionally, the role of BK_Ca_ in MT in cirrhosis will require additional in-depth investigation. And while we observed changes in ROCK2 expression, we did not assess the expression of other Ca^2+^ sensitizers such as PKC. Despite these limitations our data comprehensively demonstrate that in cirrhosis, multiple adaptations are responsible for reduced vascular tone, and targeting only one component such as enhancing Ca^2+^ influx, or modulating ROCK2 expression may not be sufficient to reverse them.

While other investigators assessed the changes in vascular tone and structural changes in superior mesenteric artery and mesenteric artery respectively in cirrhotic and portal hypertensive rats, our study provides additional insights into the mechanisms underlying changes in vascular tone, which are as follows: 1) We conducted all the experiments in pressurized small mesenteric arteries that *developed spontaneous vasoconstriction*, which is the most relevant physiological model; 2) We have demonstrated for the first time that in cirrhosis, pressure-mediated vascular tone is reduced. None of the previous studies investigated the changes in pressure-mediated vascular tone, the key contributor of SVR; 3) Our data indicate that reduction in pressure-mediated vascular tone is multifactorial i.e., changes in expression and function of MMP-9, TRPC, and ROCK2; 4) By demonstrating that GTP-mediated tone is reduced in the small-mesenteric arteries, we showed that reduced ROCK2 expression is *functionally* responsible for reduced vascular tone in cirrhosis; 5) Our data suggest that circulating ROS may be responsible for regulating the expression of MMP-9 and ROCK in small mesenteric arteries, which in turn reduce pressure-mediated vascular tone; 6) Finally, we provided a proof-of-concept *in vitro* that in vascular smooth muscle cells, oxidative stress can reduce ROCK2 expression and enhance the MMP-9 activity. These novel findings enhance our insight into the mechanisms underlying reduced SVR in cirrhosis.

Repeated liver injury as seen in alcohol abuse, fatty liver diseases or chronic viral hepatitis leads to generation of ROS within hepatic parenchyma that promotes hepatocyte apoptosis, activates stellate cells (the hepatic fibroblasts) and plays a significant role in the development of fibrosis and cirrhosis. In animal models, treatment with anti-oxidants reduces chronic liver injury. Previously, investigators showed that treatment of BDL-rats with an anti-oxidant improves vascular dynamics [48]. However, and very importantly, it is not clear if such an improvement is due to a direct effect on vasculature, or a consequence of improvement in liver injury and fibrosis [28]. Here we propose that in cirrhosis, circulating ROS are likely responsible for the vascular dysfunction of small resistance arteries by inducing oxidative stress. Therefore, in future, novel anti-oxidant molecules that are effective on vasculature alone and not the liver, or can be exclusively applied to the resistance arteries without absorption in the liver, will be crucial in answering this question.

## MATERIALS AND METHODS

### Bile duct ligation

All animal experiments were performed according to the Guide for Care and Use of Laboratory Animals prepared by the United States National Academy of Sciences (National Institutes of Health) and approved by the Institutional Animal Care and Use Committee. Male *Sprague-Dawley* rats (~250 g, Harlan Laboratories, USA) were housed under identical conditions in a pathogen-free environment with a 12:12 h light: dark cycle and had free access to laboratory chow and water. Rats were acclimatized for a week before the surgery. BDL was performed as described earlier [49]. Briefly, rats were anesthetized with ketamine (100 mg/kg *i.p*.) and xylazine (4 mg/kg *i.p*.), a laparotomy performed, the bile duct isolated, doubly ligated with a 5-0 silk suture, and resected between two ligatures. The abdominal wall was closed with a 4-0 PGA suture and the skin with a 4-0 nylon suture (all sutures; AD Surgical, Sunnyvale, CA). The control animals (SHAM) underwent laparotomy, isolation of bile duct that was not ligated, and abdominal wall closure. Animals were weighed weekly. Approximately 4 weeks after the surgery, all animals were euthanized. Their blood and livers were collected and stored, and mesenteric arteries harvested.

### Solutions

All reagents were purchased from Sigma-Aldrich (St. Louis, MO) unless indicated. Dissection solution (mM/L): 3.0 MOPS, 145.0 NaCl, 5.0 KCl, 2.5 CaCl_2_, 1.0 MgSO_4_, 1.0 KH_2_PO_4_, 0.02 EDTA, 2.0 sodium pyruvate and 5.0 glucose (pH 7.4). Physiological salt saline (PSS; mM/L): 112.0 NaCl, 25.7 NaHCO_3_, 4.9 KCl, 2.0 CaCl_2_, 1.2 MgSO_4_, 1.2 KHPO_4_, 11.5 glucose and 10.0 HEPES. High relaxing (HR) solution (pCa9.0) in mM: 53.28 KCl, 6.81 MgCl_2_, 0.025 CaCl_2_, 10.0 EGTA, 5.4 Na_2_ATP, and 12.0 creatine phosphate. The composition of pCa4.5 solution was similar to HR, except the following (mM/L): 33.74 KCl, 6.48 MgCl_2_, and 9.96 CaCl_2_. [Ca^2+^] were achieved by mixing different volumes of HR and pCa4.5 solutions. The permeabilization solution contained protease inhibitors leupeptin (1 μg/ml), pepstatin A (2.5 μg/ml), and phenylmethanesulfonylfluoride (50 μM).

### Pressure myography

Fourth-order mesenteric arteries parallel to the small intestine were dissected. Three-five mm arterial segments were transferred to a perfusion chamber, cannulated and pressurized as described previously [12, 13]. The chamber was transferred to an inverted microscope (Motic AE31, Canada), and superfused with warm PSS (2 ml/min, 37^ᵒ^C, pH 7.4, equilibrated with gas mixture: 5% CO_2_, 5% O_2_ and 90% N). The intraluminal pressure was increased to 100 mmHg, and segments with leaks were discarded. Subsequently, arterial segments were allowed to equilibrate at 70 mmHg [12]. The arteries were viewed with a 10X objective equipped with a monochrome video charge-coupled device camera, and their luminal diameter recorded continuously by image capture with a video frame grabber and real-time edge-detection system (Ionoptix, Milton, MA). At the end of each experiment, passive diameter (PD, i.e., maximal diameter) was determined by incubating the arteries in Ca^2+^-free PSS for 20 min. All experiments were performed at 70 mmHg unless indicated.

### Myogenic response

The arteries were subjected to incremental pressures between 10 and 110 mmHg (10, 30, 50, 70, 90 and 110), and at each pressure-step, diameter was allowed to stabilize for 5 min. Subsequently, the pressure-response curve was repeated in Ca^2+^-free PSS (containing 3 mM EGTA and 0.01 mM diltiazem). For each pressure-step, MT was calculated as the percent difference in diameter with and without Ca^2+^ containing PSS [12].

The intraluminal cross sectional area was calculated as πr^2^. The circumferential stress was calculated as: (P*d*i)/(2*M*) where P was intraluminal pressure (mmHg), *d*i lumen diameter (μm), and *M* wall thickness (μm). The circumferential strain was calculated as: (*d* - *d*_10_)/*d*_10_ where *d* was stable lumen diameter at an intraluminal pressure-step, and *d*_10_ was diameter at 10 mmHg.

### Arterial permeabilization

The arteries were incubated in 50 μM β-escin in HR solution [41, 50–52]. After 1 h, the arteries were washed with HR solution and pressurized at 70 mmHg. Ca^2+^ sensitivity was assessed by superfusing the permeabilized arteries with solutions of varied [Ca^2+^] (pCa9.0, 4.5 or 7.5).

### Quantitative PCR (qPCR)

The mesenteric arterial RNA was extracted using RNeasy Mini Kit (Qiagen, Valencia, CA). cDNA was prepared from 0.5 μg total RNA/reaction using iScript cDNA synthesis kit (Bio-Rad, Hercules, CA) and diluted with the nuclease-free water. 25 μl reaction volume comprised 12.5 μl Quantifast SYBR green master mix (2X), 1 μl cDNA (25 ng), 1 μl primer (10 pmol/μl each), and 9.5 μl nuclease-free water. A two-step thermal cycling profile was used for amplification (StepOnePlus Real-Time PCR System, Applied Biosystems, Life Technologies, Grand Island, NY). Step I (cycling): 95 °C for 5 min, 95 °C for 10 s and 60 °C for 30 s for 40 cycles. Step II (melting curve): 60 °C for 15 s, 60 °C 1 min and 95 °C for 30 s. The specific template amplification was confirmed by melting curve analysis. mRNA fold change was calculated using ΔΔCT method by normalizing data with housekeeping (GAPDH) gene expression. Respective forward and reverse primer sequences for target genes are shown in Table 1.

**Table 1:** primer sequences

Gene	Primer sequences	
	Forward	Reverse
BKCa	TCAGCATTGGTGCCCTTGTA	CTGCAATAAACCGCAAGCCA
Cav1.2	ATGAGCATGCCCACAAGTGA	AGTAGCGGCTGAACTTGGAT
eNOS	GGCTGAGTACCCAAGCTGAG	ATTGTGGCTCGGGTGGATTT
Gapdh	TCTCTGCTCCTCCCTGTTCTA	TACGGCCAAATCCGTTCACA
iNOS	TGTTAGCCTAGTCAACTACAAGC	GTTGTTGGGCTGGGAATAG
nNOS	AAGGTCCGATTCAACAGCGT	CCGAACACTGAGAACCTCACA
TRPC6	TATCTGCTGATGGACGAGCTG	GTTTTCATTACCCCGGAAGCTG

### Immunoblotting

Four-to-five snap-frozen arterial segments from same animal were homogenized in RIPA lysis buffer (containing protease and phosphatase inhibitors), kept on ice for 2 h, then centrifuged at 22000 g for 20 min (4°C). The supernatant was collected and protein concentration determined using Bradford assay (Sigma-Aldrich, St. Louis, MO). Equivalent protein content was electrophoresed in polyacrylamide gels and transferred onto polyvinylidene-fluoride membranes. The membranes were probed with primary antibodies to ROCK2, RhoA, pMLC, MLC, (all 1:1000, Cell Signaling, Danvers, MA), MMP9 (1:1000, Santa Cruz Biotechnology, Dallas, TX), eNOS (1:1000, BD Bioscience, San Jose, CA), Collagen-1 (1:500, Southern Biotech, Birmingham, AL), Ca_v_1.2, BK_Ca_, TRPC6 and TRPC3 (all 1:500, Alomone Labs, Israel) overnight at 4°C, followed by anti-rabbit (1:3000, Cell Signaling) or anti-goat (1:3000, Sigma Aldrich, St. Louis, MO) IgG-HRP secondary antibodies. The blots were developed using Clarity Western ECL Substrate (Bio-Rad, Hercules, CA) and autoradiography films (Genesee Scientific, San Diego, CA). The membranes were stripped using a tris-buffered solution with 2% sodium dodecyl sulfate and 100 mM β-mercaptoethanol at 50°C, and re-probed with different antibodies. Band intensities were quantified by NIH Image J (http://imagej.nih.gov/ij/) and normalized to β-actin. All original blots are provided in the supplementary file.

### Histology and immunostaining

The paraffin-embedded arterial sections were de-paraffinized with xylene, and rehydrated with series of ethanol gradient. Following antigen retrieval (using citric acid buffer), the sections were incubated in 2% H_2_O_2_ for 30 min to block endogenous peroxidase activity. To block non-specific protein binding, sections were washed with PBS and treated with 1% BSA (60 min) and 5% goat serum (30 min). Then, the sections were incubated with anti-TRPC6 or anti-BK_Ca_ polyclonal antibodies (1:200, Alomone Labs, Israel) overnight at 4°C in a humidified chamber. The following day, the sections were washed with PBS and incubated with biotinylated goat anti-rabbit IgG (H+L) secondary antibody for 60 min at room temperature. The avidin-biotin reaction was carried out using VECTASTAIN Elite ABC kit (Vector Laboratories, Burlingame, CA) and the sections were treated with diaminobenzidine (Vector Labs). The negative controls were processed similarly but without incubation in primary antibodies. Finally, the sections were counterstained with hematoxylin, mounted using DPX, and examined under a microscope.

### Cell culture

The SV40LT-transfected rat VSM cells (VSMCs, ATCC, USA) were grown in DMEM containing 10% FBS and antibiotics, and sub-cultured using 0.25% (w/v) Trypsin-0.53mM EDTA solution.

### Zymography

Gelatin zymography was performed as described previously [53]. VSMCs (1.0 × 10^6^) were treated with peroxynitrite (2.5 μM) or its vehicle NaOH (0.5 mM), and SIN-1 (2.5 μM) or its vehicle DMSO (0.05%) in serum-free DMEM. After 24 h, 500 μl culture media was collected, concentrated using Amico Ultra 0.5 ml centrifugal filters (Merck Millipore, Billerica, MA), loaded (7.5 μl/well) onto 8% SDS-polyacrylamide-gelatin gels and analyzed for MMP-9 activity. Similarly, equivalent amount of arterial protein (30 μg) was electrophoresed under non-reducing conditions. The gels were washed with 2.5% Triton X-100 for 45 min, placed for 48 h in incubation buffer (50 mM Tris-HCl, 150 mM NaCl, 5 mM CaCl_2_, and 0.05% NaN_3_) at 37°C, stained with 0.05% Coomassie Brilliant Blue, and then de-stained until enzyme activity was detected as colorless bands and photographed in a gel documentation system (Bio-Rad, Hercules, CA, USA). Band intensities were quantified by NIH Image J (http://imagej.nih.gov/ij/). A protein ladder (Bio-Rad, Hercules, CA) was used to identify MMP-9. All original blots are provided in the supplementary file.

### Measurement of plasma ROS

The rat plasma was incubated with 100 μM dichlorofluorescein diacetate (DCFDA; Invitrogen, Carlsbad, CA, USA) in dark for 10 min at 37°C. Fluorescence intensity was recorded using excitation and emission wavelengths of 485/520 nm in a Synergy 2 Multi-Mode Microplate Reader (Bio-Tek, Winooski, VT). The plasma malondialdehyde (MDA), a lipid peroxidation product, was quantified by measuring thiobarbituric acid reactive substances (TBARS) formation as described previously [54]. 1, 1, 3, 3-tetraethoxypropane was used as the standard to calculate MDA content (nmoles/ml).

### Statistical analysis

The results are presented as mean ± S.E.M. Statistical significance was defined as *p* ≤ 0.05 and determined using the student's *t*-test (normally-distributed data) and the Mann-Whitney U-test (nonparametric data).

## SUPPLEMENTARY MATERIALS FIGURES


