# Supplementary material for: Characterization of pressure-mediated vascular tone in resistance arteries from bile duct-ligated rats
Source: Oncotarget. 2017 Feb 16;8(19):30706–22. doi: 10.18632/oncotarget.15409 (PMC5458161; doi:10.18632/oncotarget.15409)

Supplementary Information

Title: Characterization of Pressure-Mediated Vascular Tone in Cirrhotic Rats

Authors: Ravirajsinh N. Jadeja[1], Menaka C. Thounaojam[2], Sandeep Khurana[1].
Affiliation: [1]Digestive Health Center, Medical College of Georgia, Augusta, GA, 30912, USA; [2]Department of Biochemistry and Cancer Biology, School of Medicine, Meharry Medical College, Nashville, TN, USA.

Correspondence: Sandeep Khurana, M.B.B.S**.**

E-mail: skhurana@geisinger.edu

Current Address: 100 N Academy Avenue

Danville, PA 17822

Ph: 570-271-6405

Fax: 570-271-6852

SFigure 1. Bile duct ligation in rats induces cirrhosis. **(A-B)** Compared to SHAM-rats, the BDL-rats gained less weight (n=7/group). One week after surgeries, the BDL-rats weighed less than the SHAM-rats. By 2nd week, the BDL-rats started to gain weight, however, during the study period their weight remained less than the SHAM-rats. At euthanasia, presence of ascites was recorded and the liver surface was inspected and graded as nodular or smooth. Almost all BDL-rats developed **(C)** ascites and **(D)** nodular liver, while the SHAM-rats had normal livers and no ascites (n=19-21/gp.). The harvested livers were fixed, paraffin-embedded and their sections stained with hematoxylin &eosin at local core facility. **(E)** Representative H&E-stained liver sections indicate that the BDL-rats had cirrhotic livers with extensive ductular proliferation. In the liver section from SHAM-rats, the bile duct is indicated by an arrow head. In the liver section from BDL-rat, among many bile ductules, some are indicated by the asterisks in their lumen. These data validated that the BDL-rats had cirrhosis at euthanasia. * $P < 0.05$, ** $P < 0.01$, and *** $P < 0.001$ when compared to SHAM-rats.

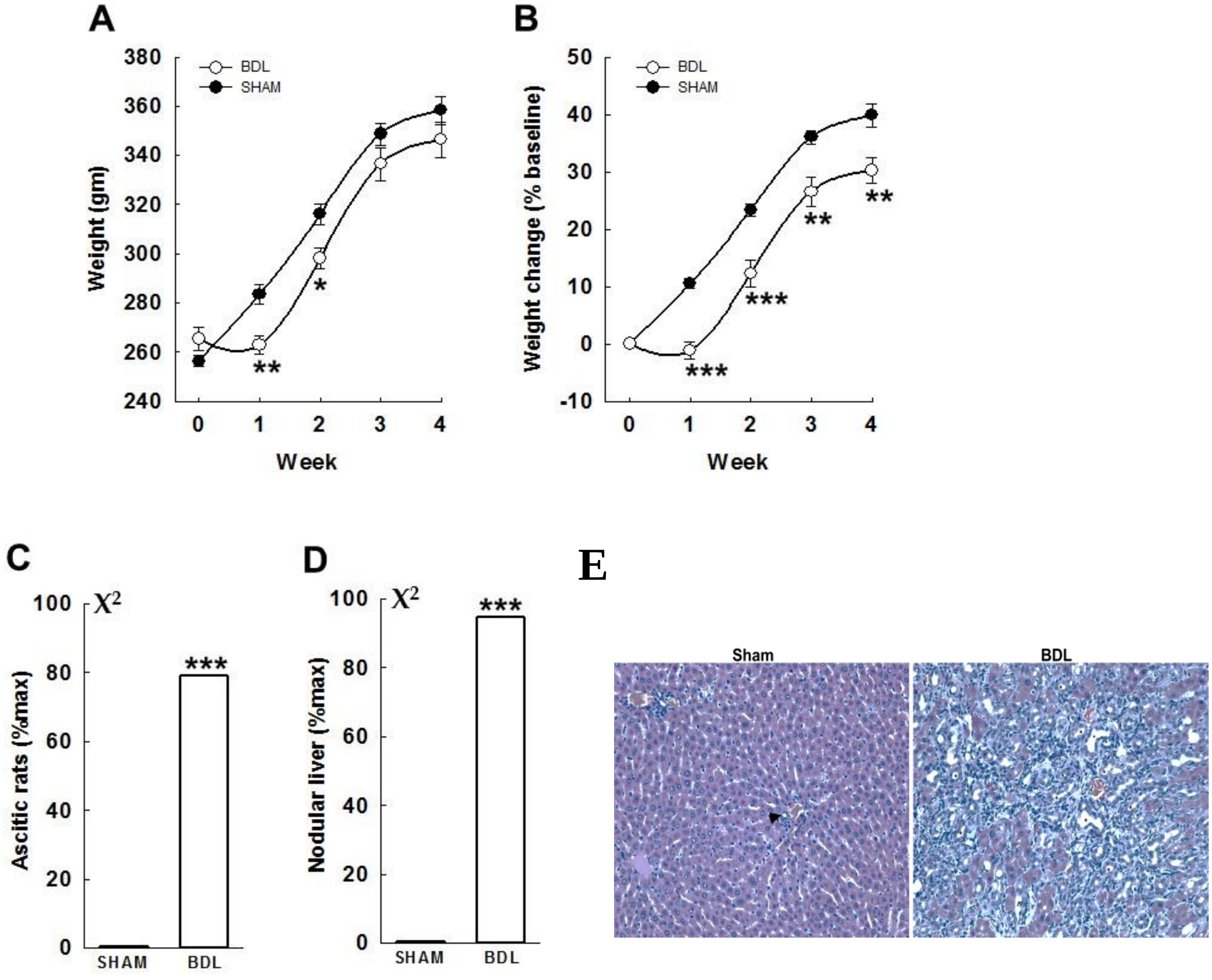

Full-length Immunoblots for Figure 2E.

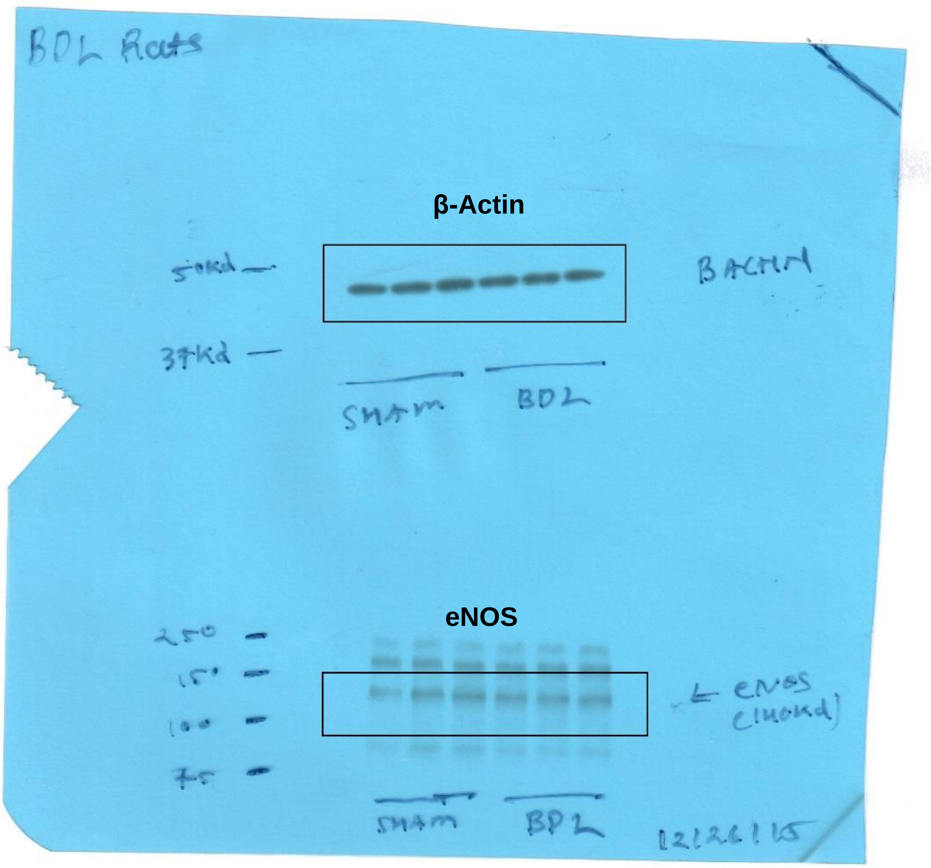

Full-length Immunoblots for Figure 4A.

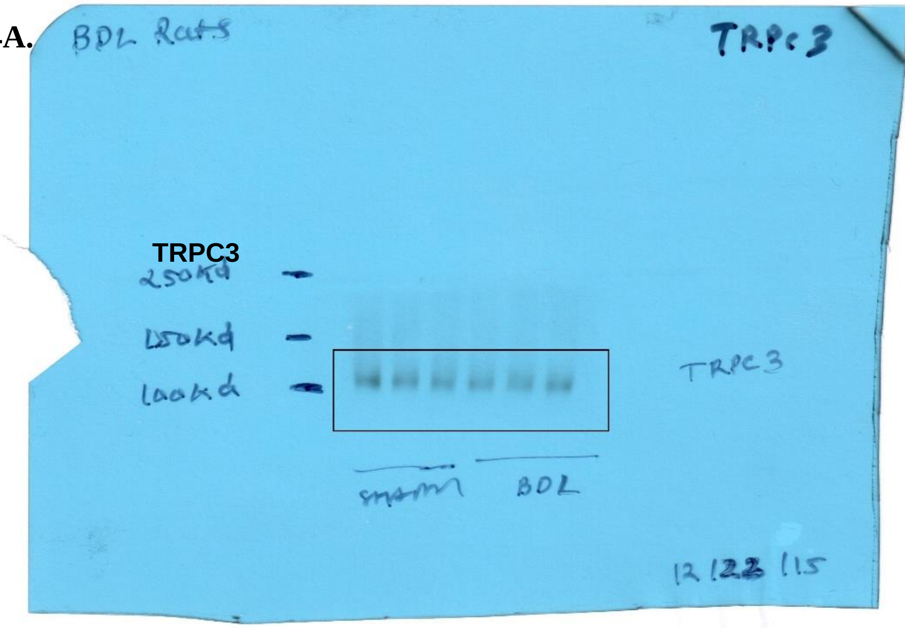

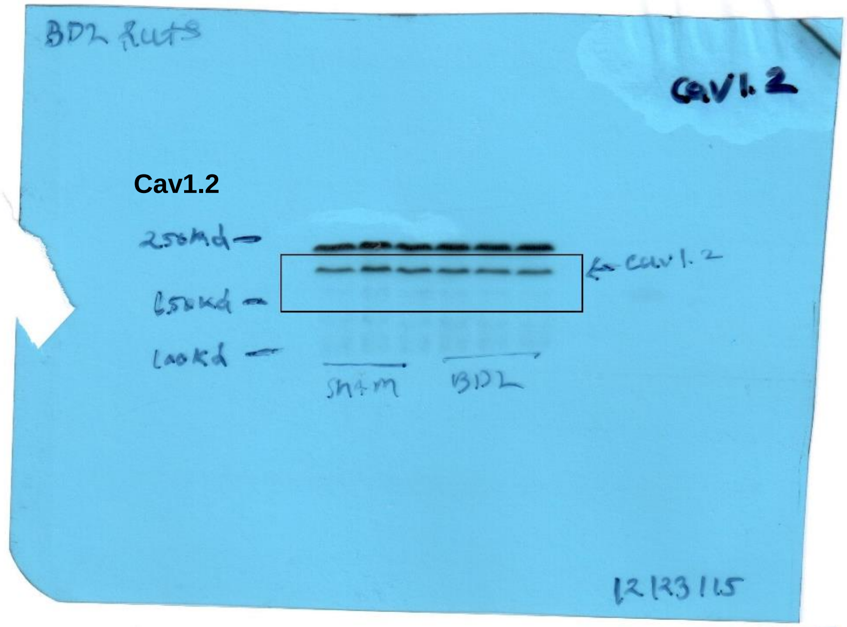

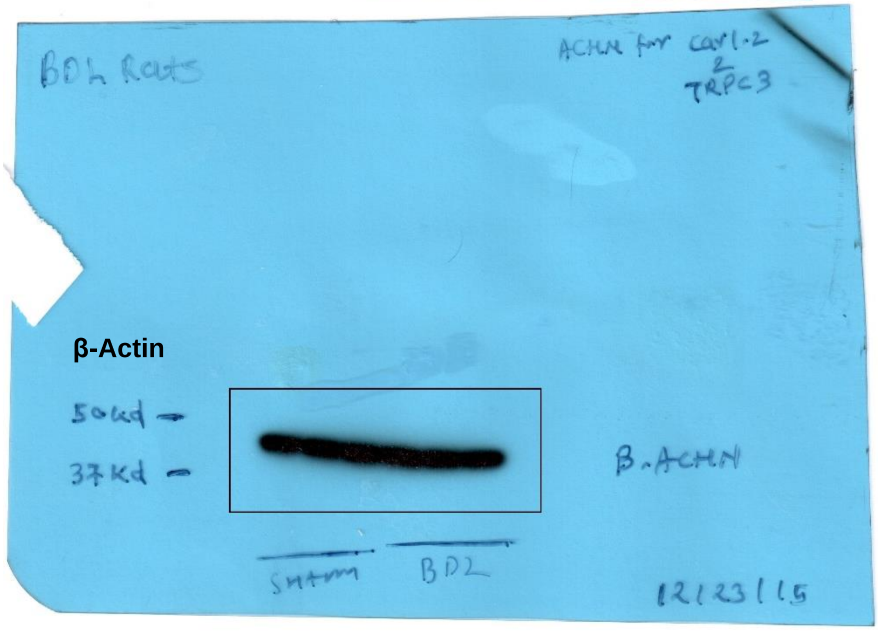

Full-length Immunoblots for Figure 4G.

TRPC6

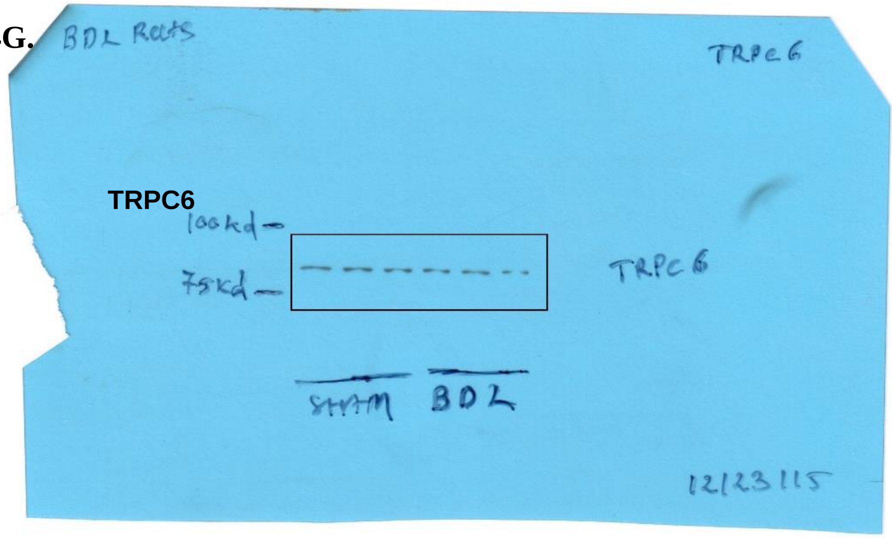

BKCa

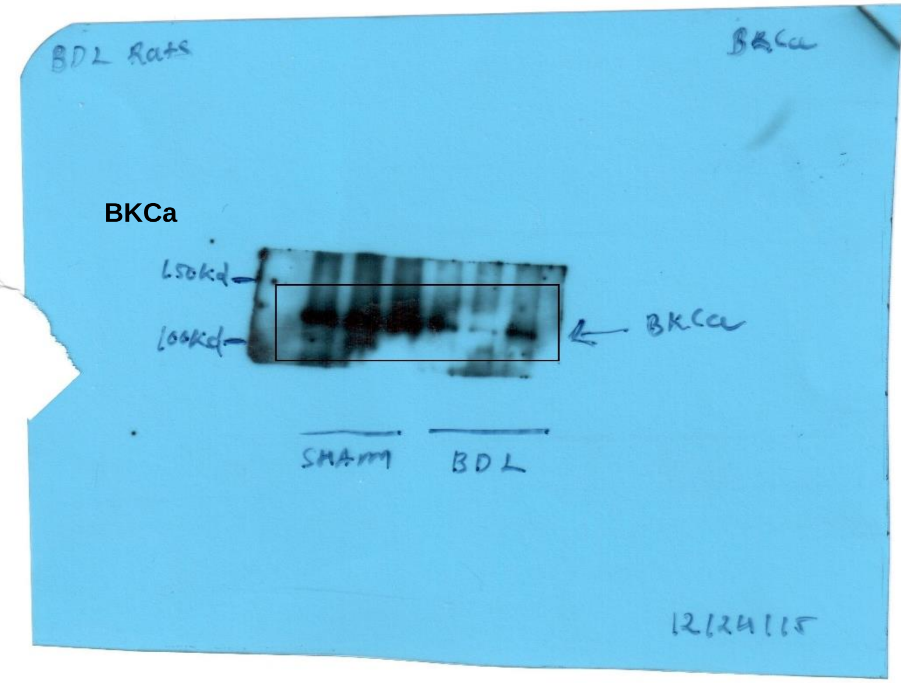

β-Actin

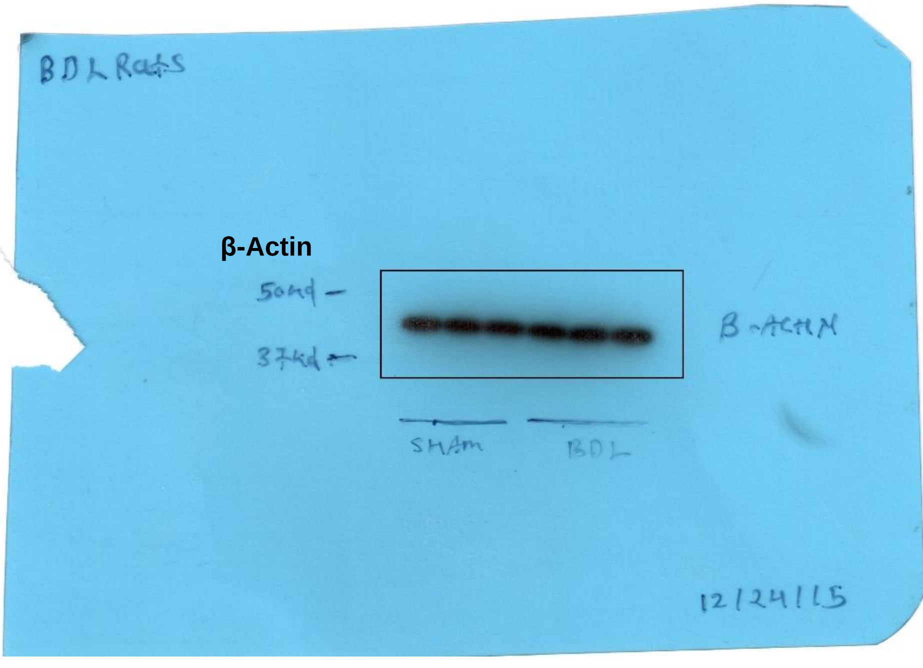

Full-length Immunoblots for Figure 5C. Replicate 1 for ROCK2.

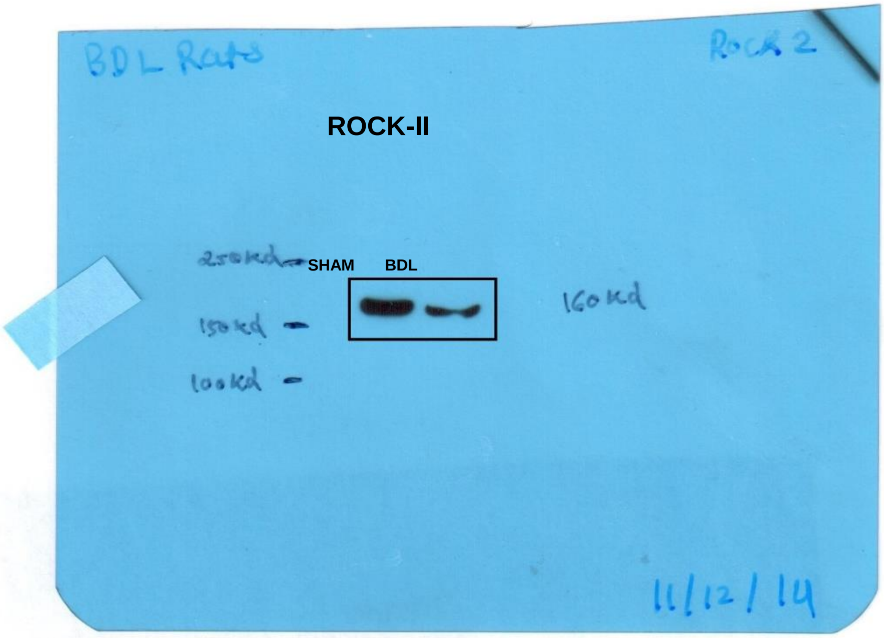

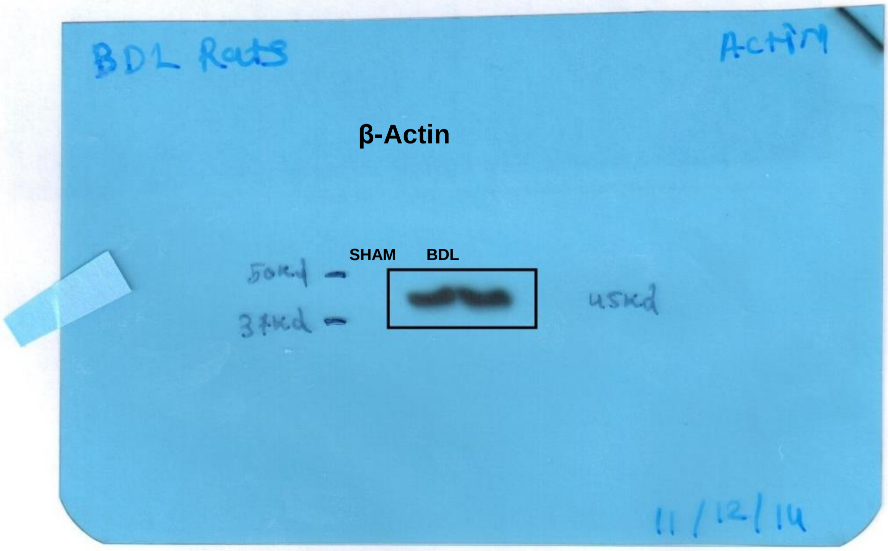

Full-length Immunoblots for Figure 5C. Replicate 2 for ROCK2.

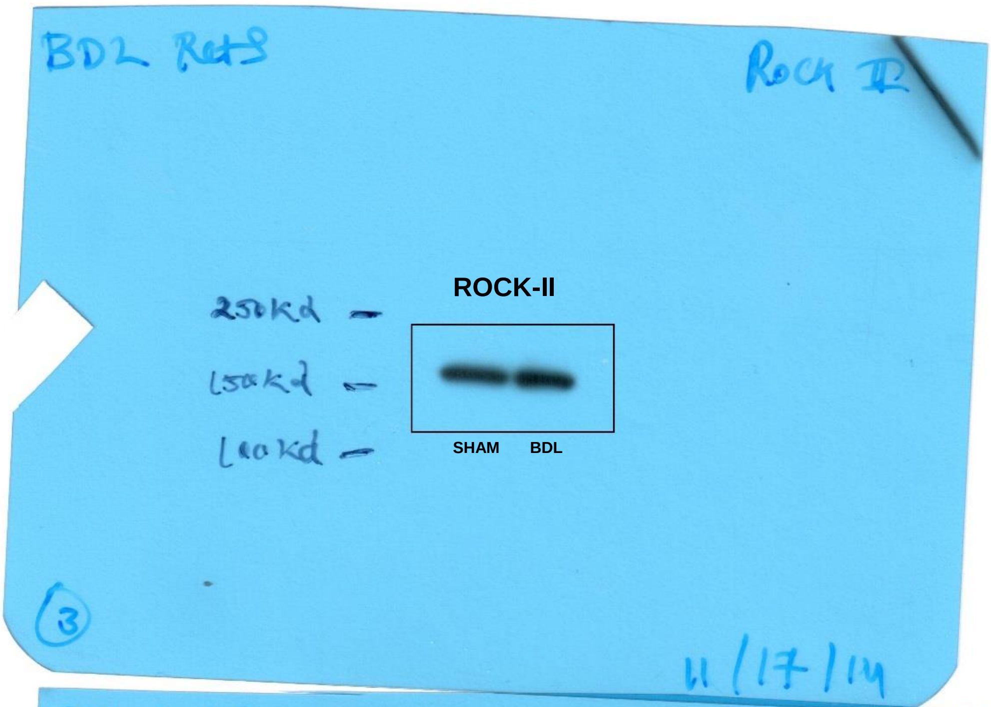

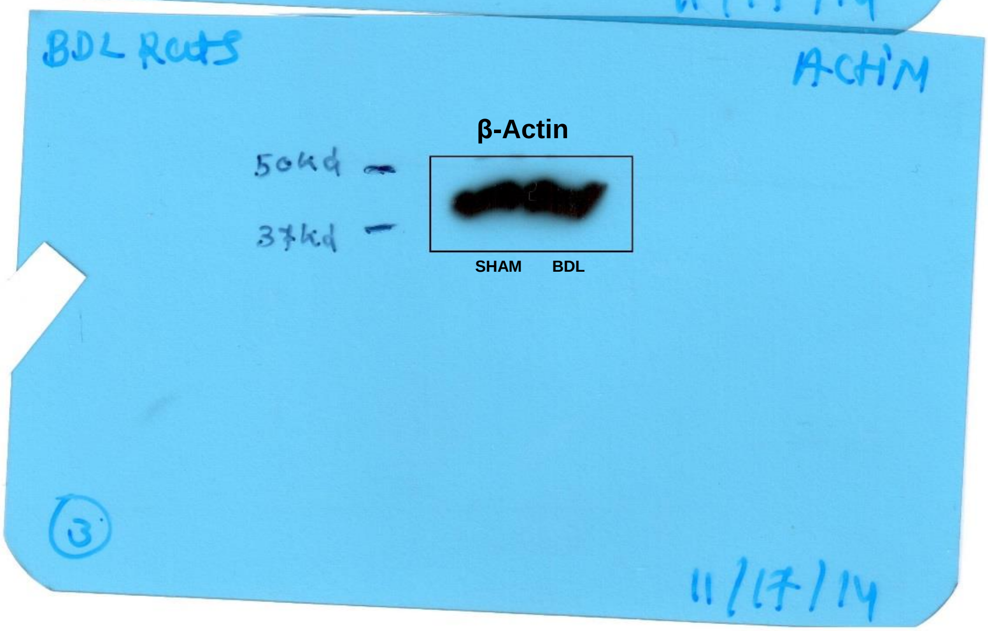

Full-length Immunoblots for Figure 5C. Replicate 3 for ROCK2.

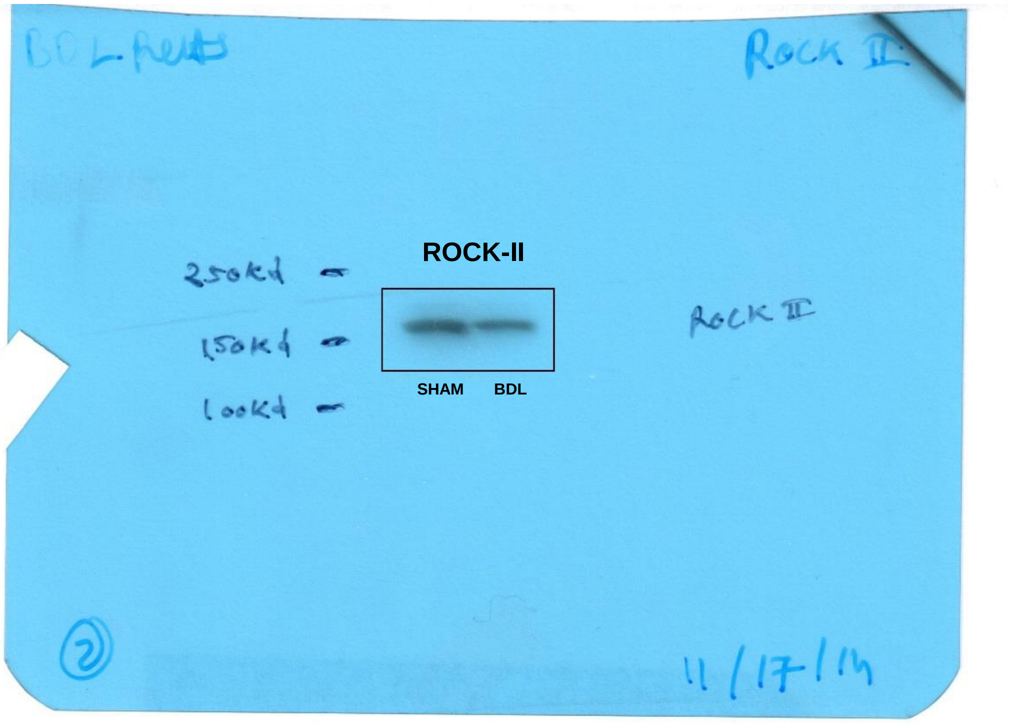

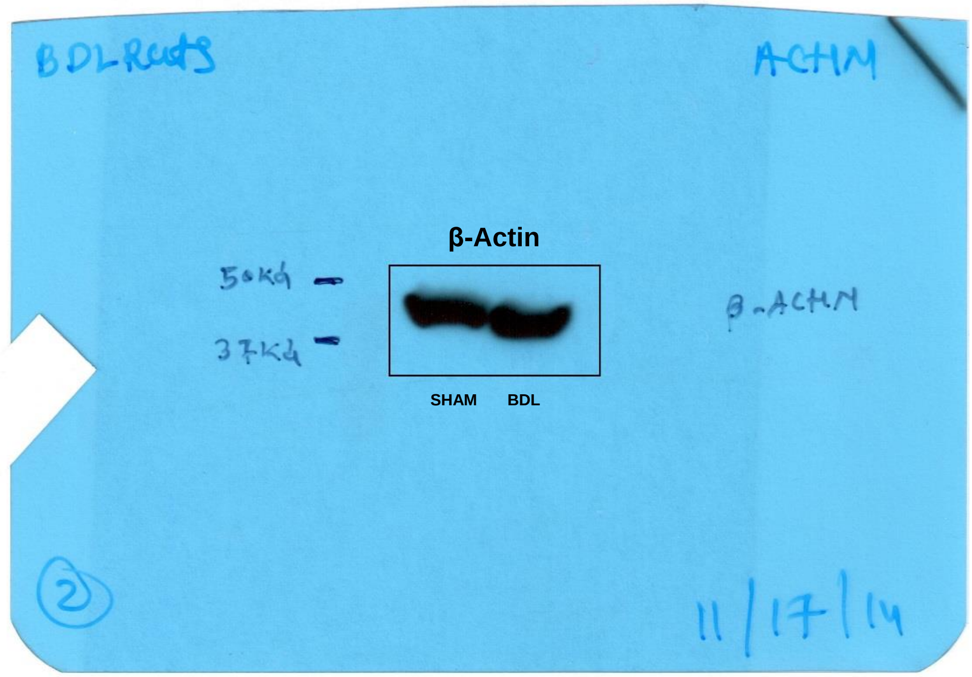

Full-length Immunoblots for Figure 5C. Replicate 1 for RhoA.

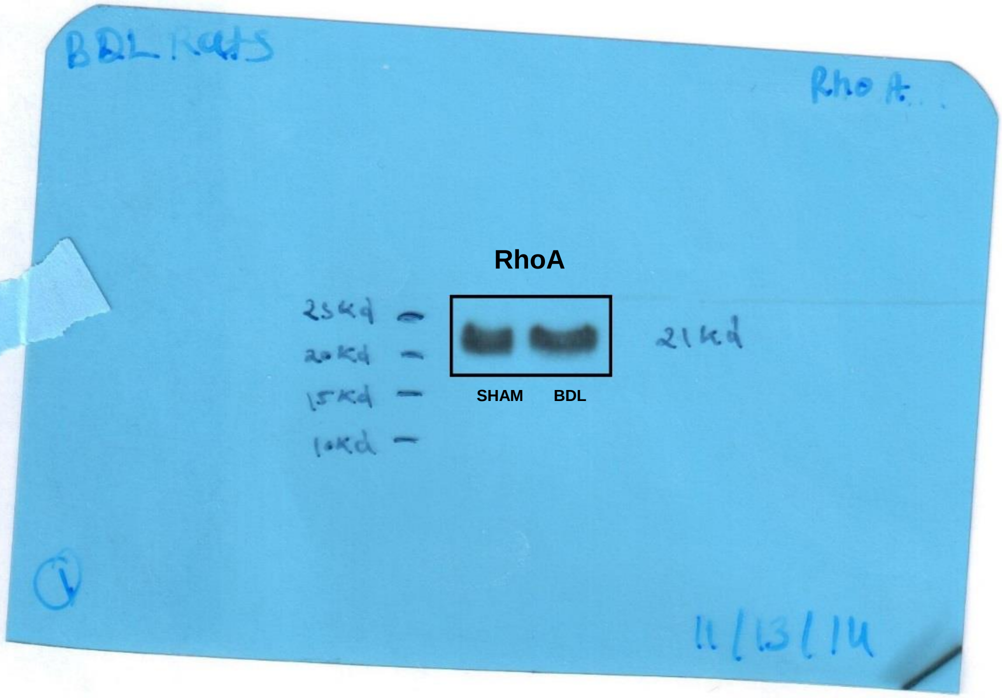

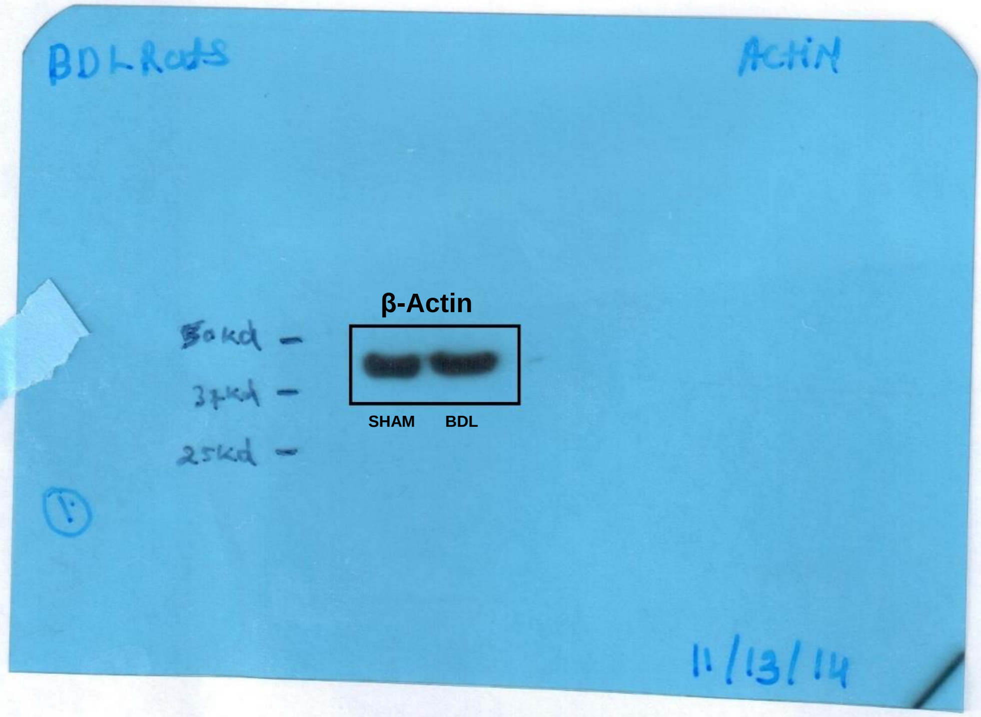

Full-length Immunoblots for Figure 5C. Replicates 2 and 3 for RhoA.

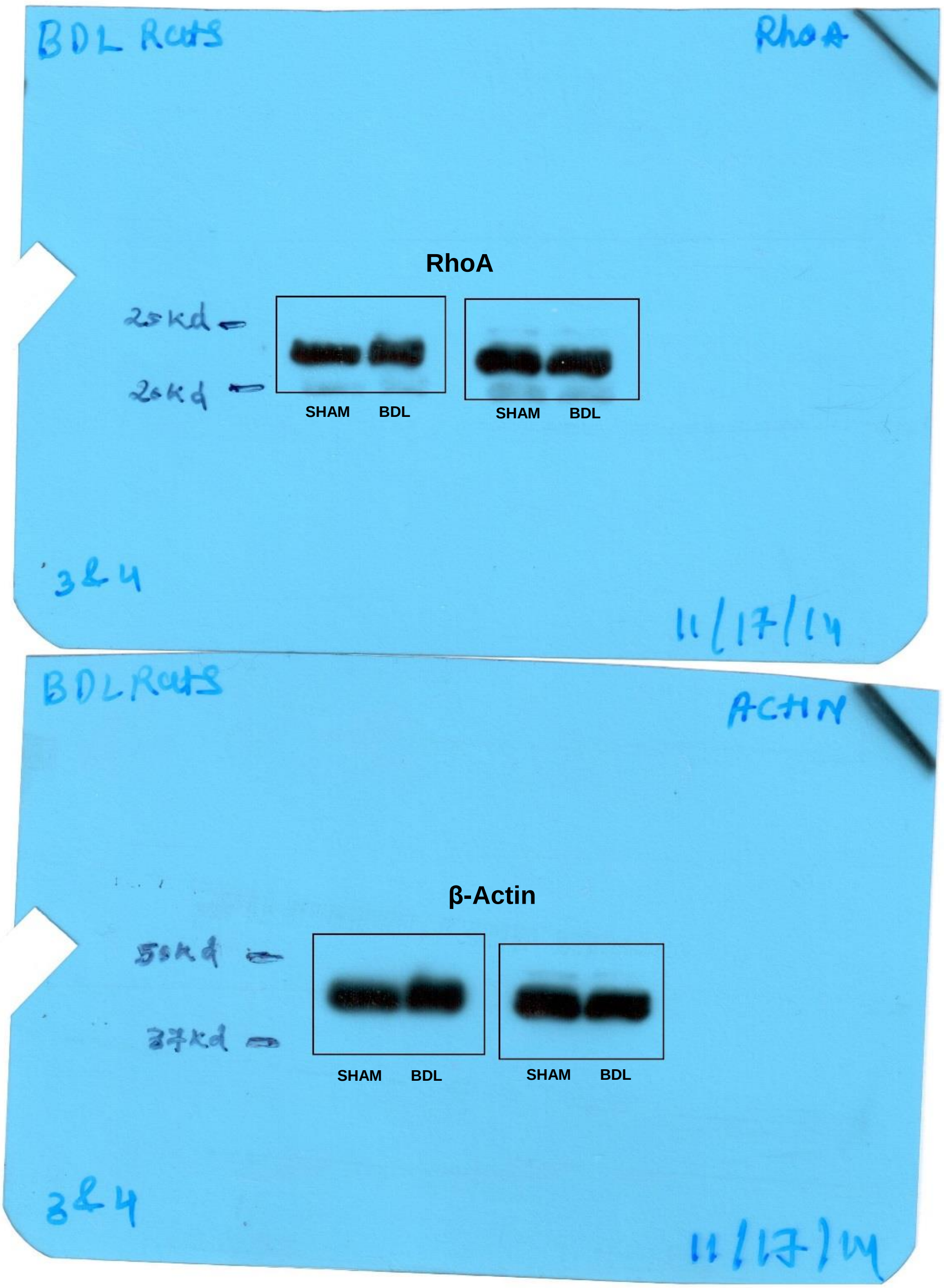

Full-length Immunoblots for figure 5C. Replicate 1 for p-MLC.

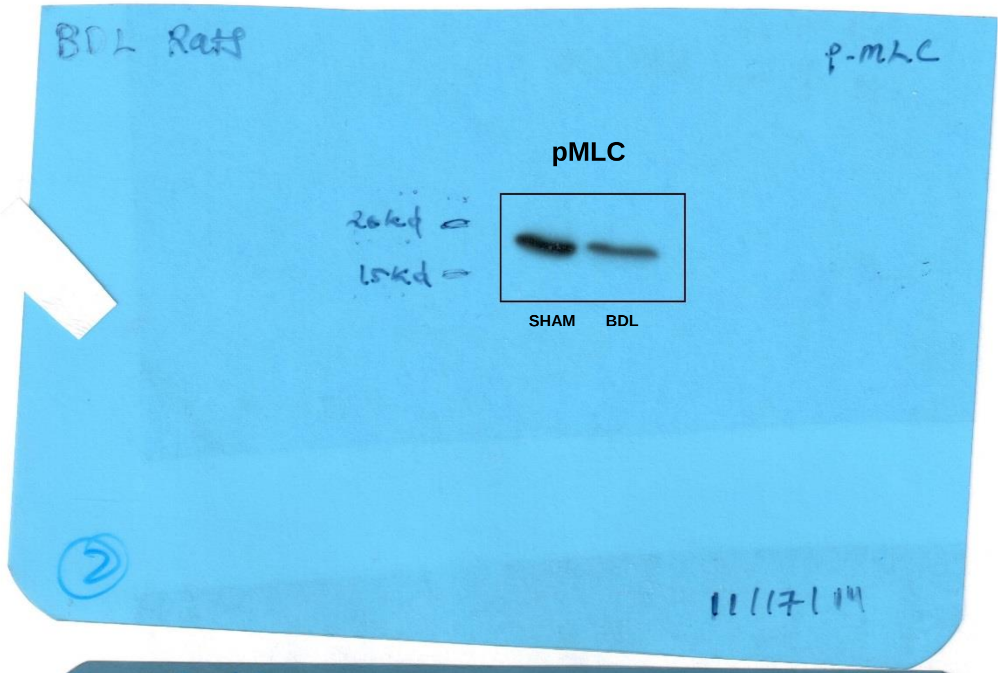

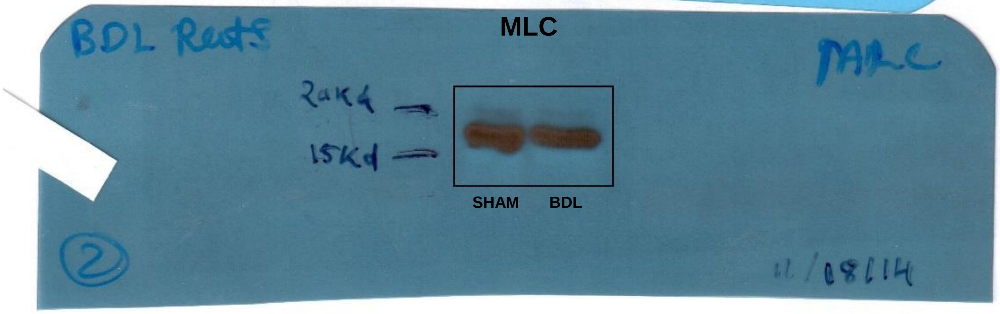

Full-length Immunoblots for figure 5C. Replicate 2 for p-MLC.

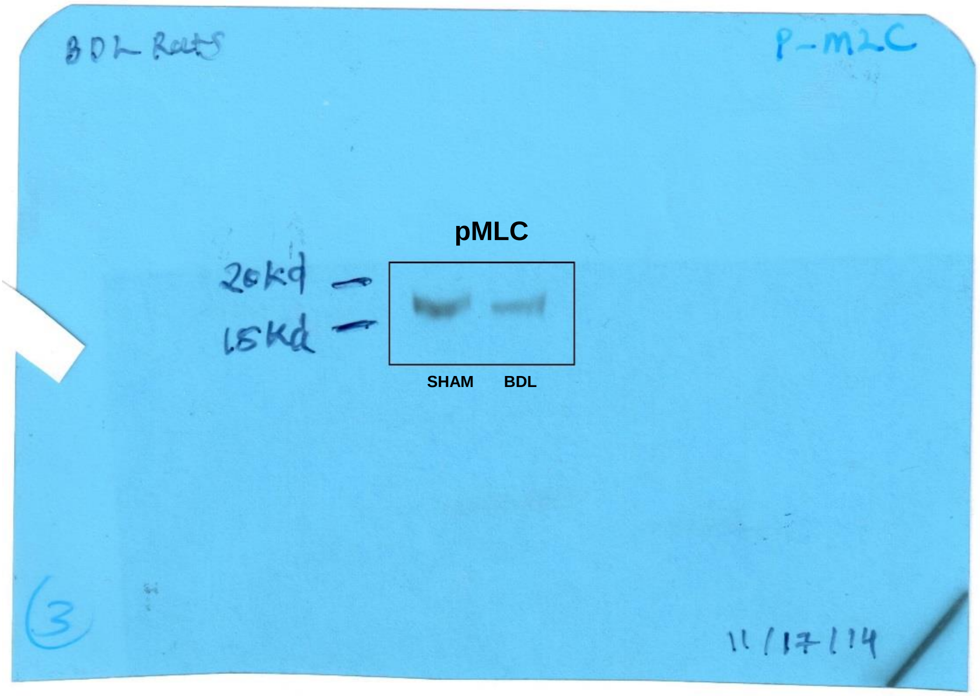

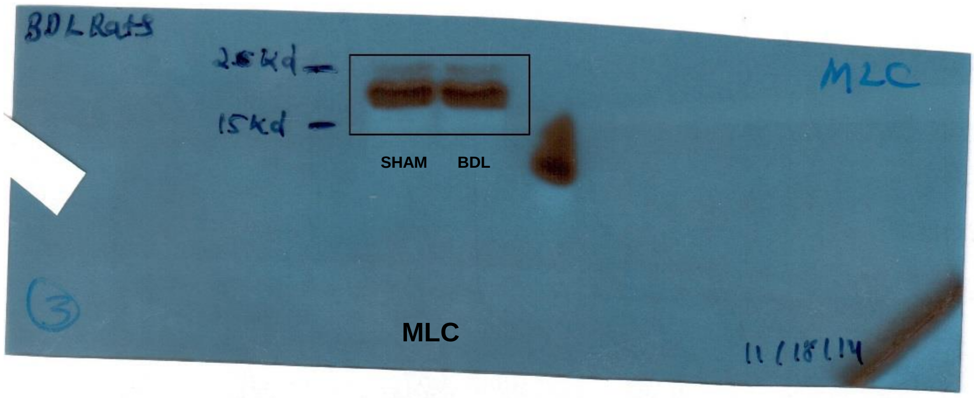

Full-length Immunoblots for figure 5C. Replicate 3 for p-MLC.

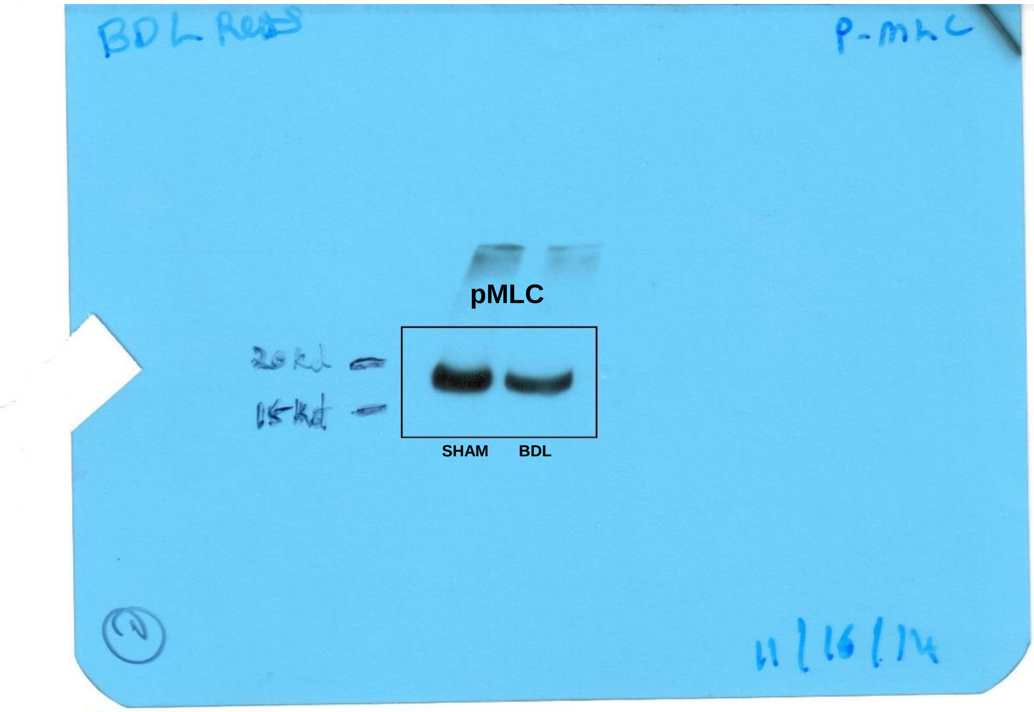

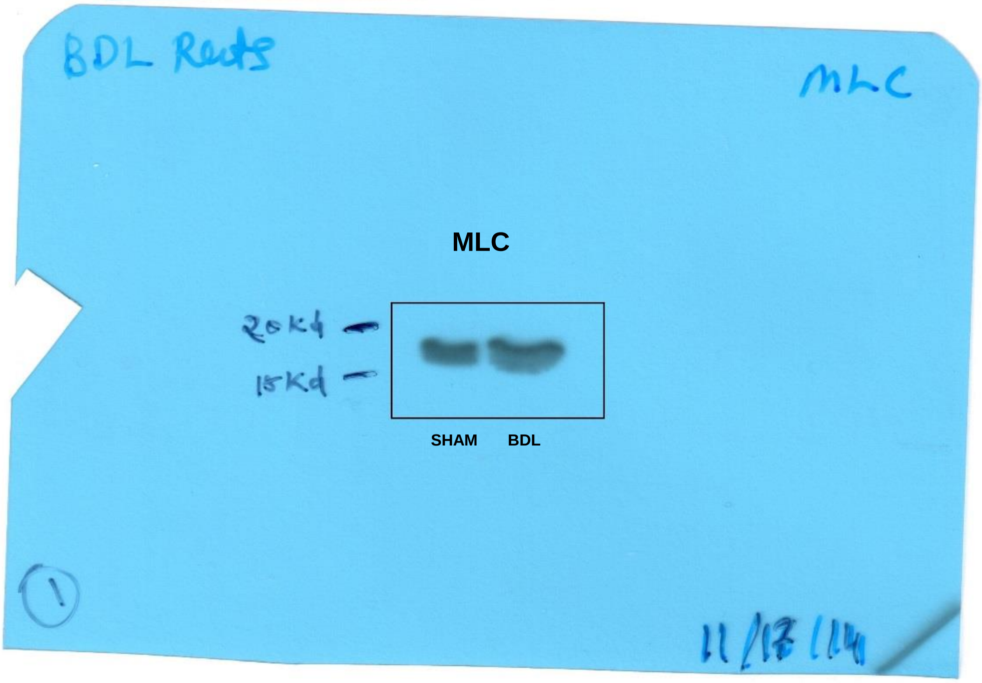

Full-length Immunoblots for Figure 6K.

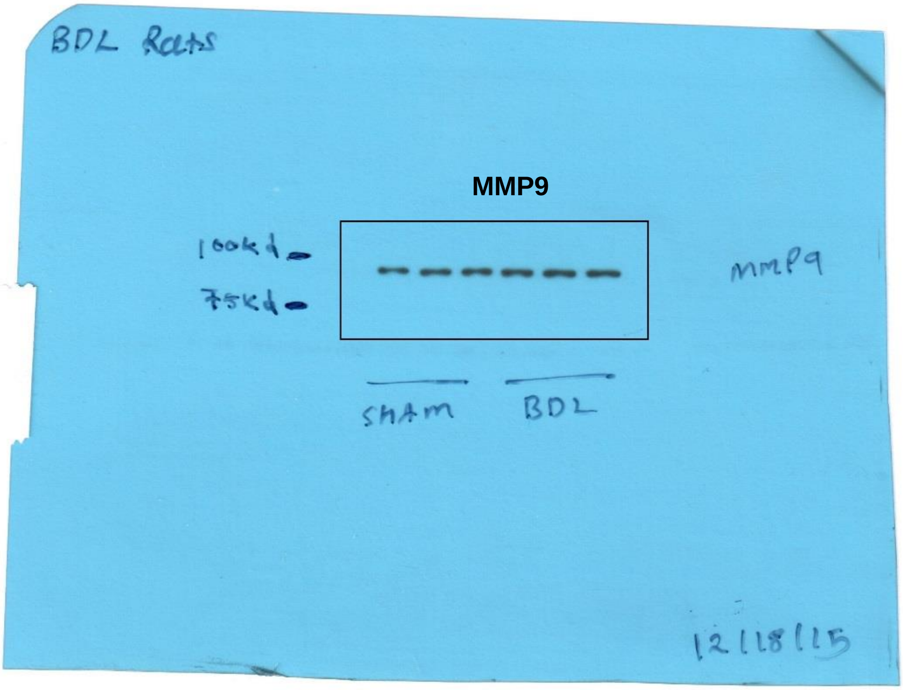

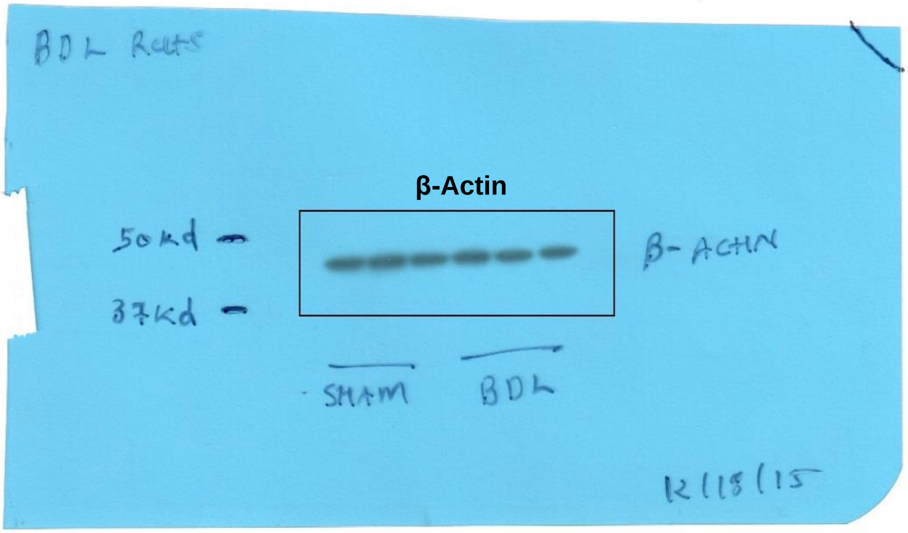

Full-length Immunoblots for Figure 6K.

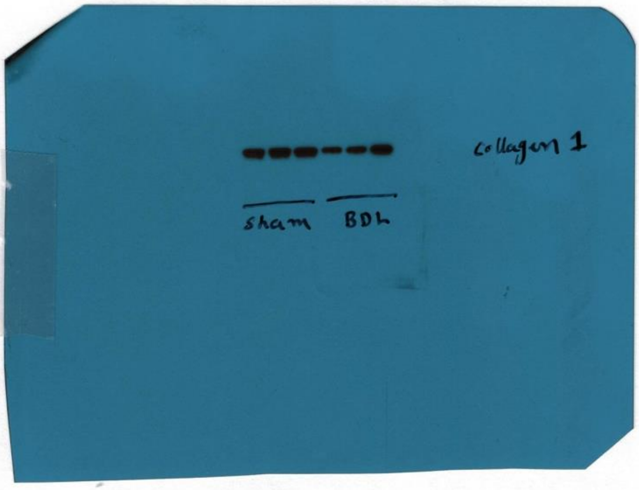

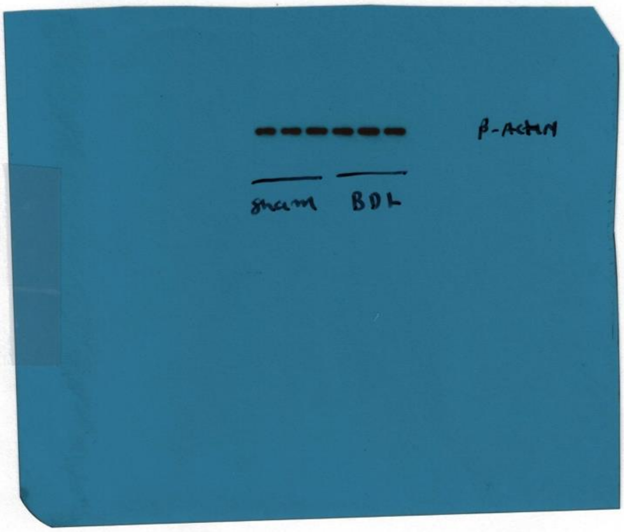

Full-length Immunoblots for Figure 7G.

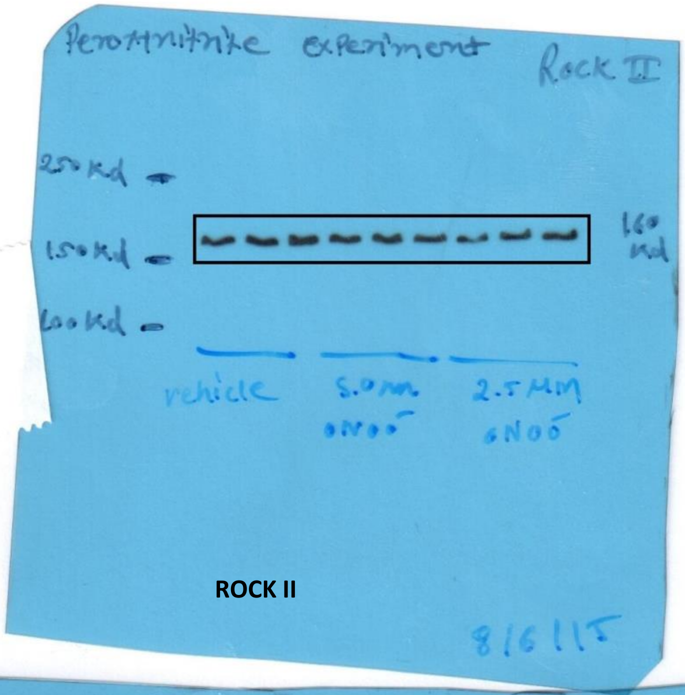

ROCK II

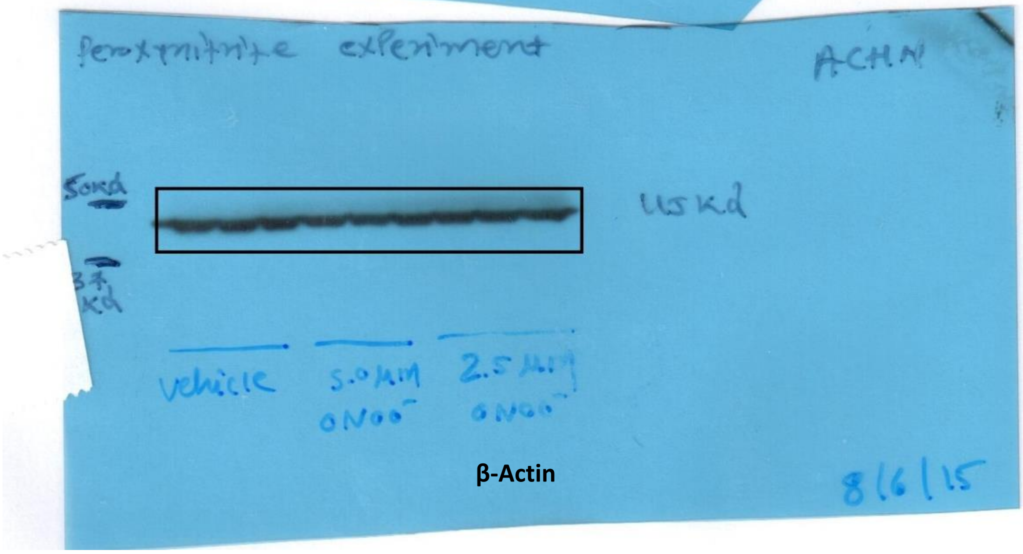

β-Actin

Full-length gelatin zymography gel for Figure 7A.

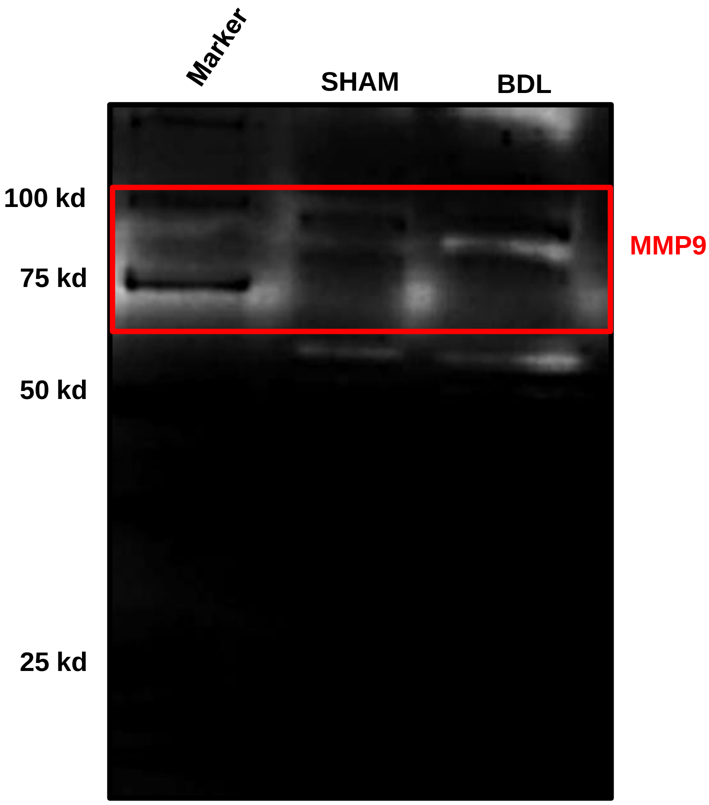

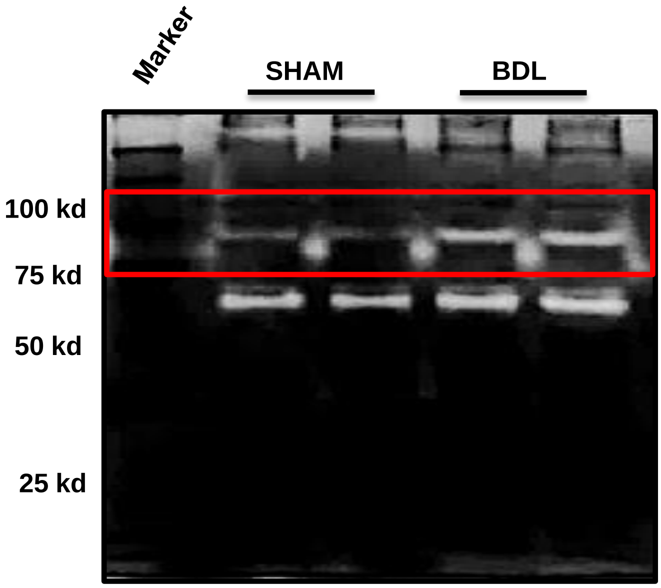

β-actin control for Zymography for arterial tissue used above

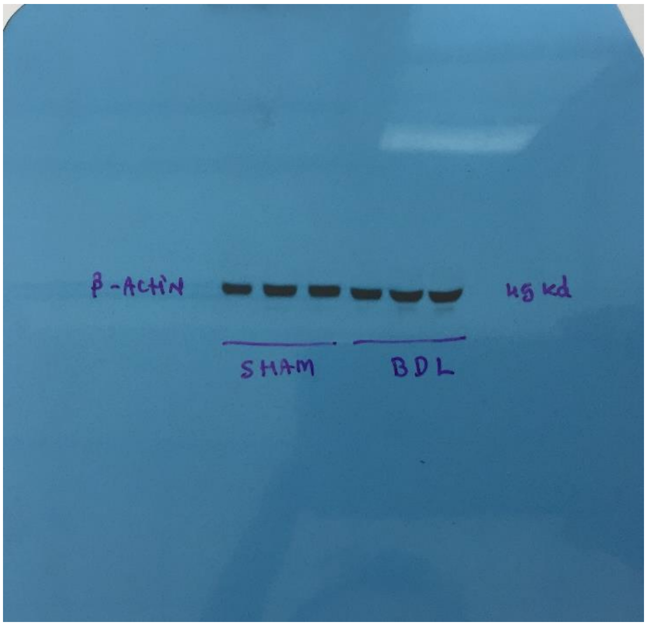

Full-length gelatin zymography gel for Figure 7E.

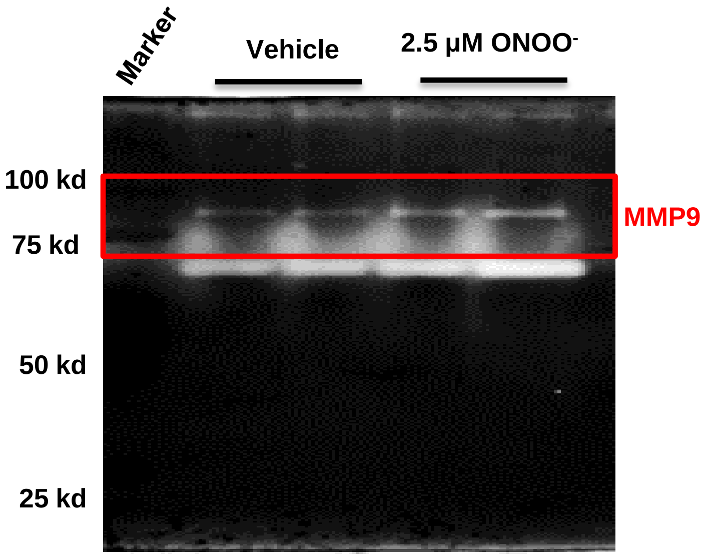

Full-length gelatin zymography gel for Figure 7F.

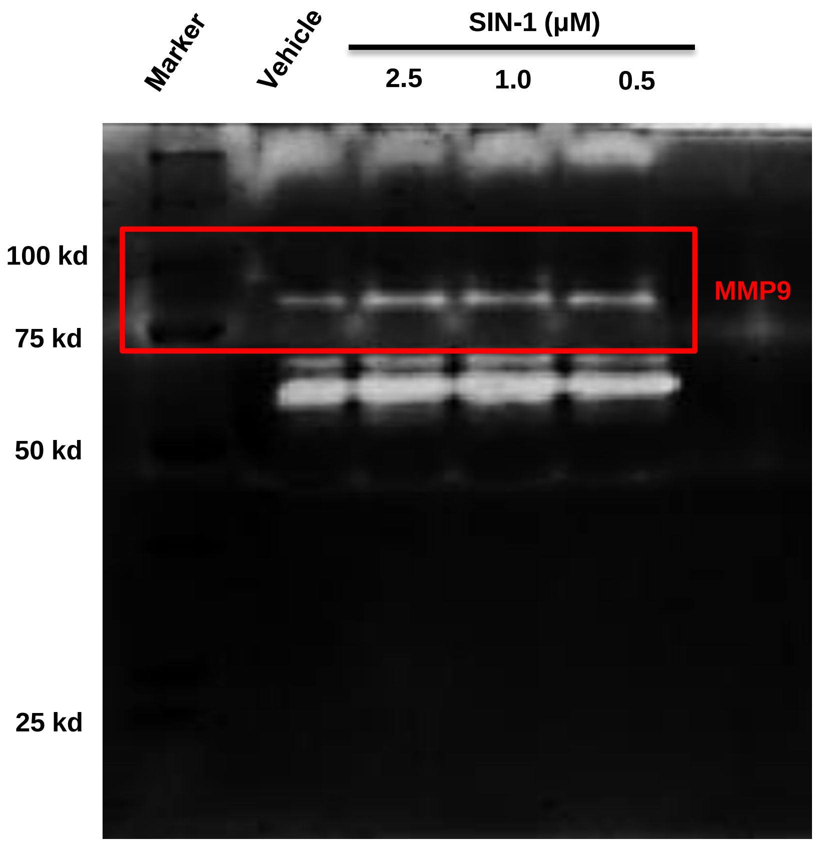

Supplement: Supplementary file 1 [file oncotarget-08-30706-s001.pdf]
